# Optimization of the Mechanical Response in MEX Additive Manufacturing of Thermoplastic Polyimide (PI): The Impact of Key Process Control Settings

**DOI:** 10.1021/acsomega.5c08277

**Published:** 2025-11-05

**Authors:** Markos Petousis, Nikolaos Mountakis, Anastasios Zavos, Ioannis Ntintakis, Amalia Moutsopoulou, Maria Spyridaki, Nektarios K. Nasikas, Emmanuel Maravelakis, Nectarios Vidakis

**Affiliations:** † Department of Mechanical Engineering, 112178 Hellenic Mediterranean University, Heraklion 71410, Greece; ‡ Department of Mechanical Engineering and Aeronautics, 37795 University of Patras, Patras 26504, Greece; § Division of Mathematics and Engineering Sciences, Department of Military Sciences, 69139 Hellenic Army Academy, 16673 Vari, Attica, Greece; ∥ Department of Electronic Engineering, 112178 Hellenic Mediterranean University, Chania 73133, Greece

## Abstract

High-performance polymers have made significant progress in the field of three-dimensional (3D) printing. An increasing number of investigations have exploited these unique properties. In this context, polyimide (PI) optimization efforts for the mechanical response of 3D printed samples were performed. Such an endeavor remains unexplored thus far because of the high processing temperature required, high material cost, and complex rheological behavior. The PI filament was extruded for the 3D printing of the specimens (material extrusion, MEX). The specimens were used for mechanical and morphological examinations. The L16 Taguchi design was employed with the raster orientation, hot-end temperature (HT), printhead velocity (PV), internal fill ratio, and deposition width as generic variable control parameters. The output metrics were the ultimate yield strength, Young’s modulus, and toughness (tensile test). Reduced quadratic regression (RQRM) and linear regression models were applied and compared. RQRM was found to be the most beneficial. The ranks indicated a significant influence of the PV parameter on the majority of the responses. HT was not highly ranked. High determination coefficients (*R* ^2^ > 0.71) enabled accurate prediction of mechanical responses (confirmation run error <10%). The optimized configuration yielded an improvement higher than 250% in all three of the tensile response metrics (230% for the fourth configuration). An experimentally validated, robust framework is provided herein for the tensile response of high-performance PI thermoplastics in MEX 3D printing, thus enabling its broader utilization in aerospace, electronics, and high-temperature tooling, in which performance and reliability are critical.

## Graphical abstract

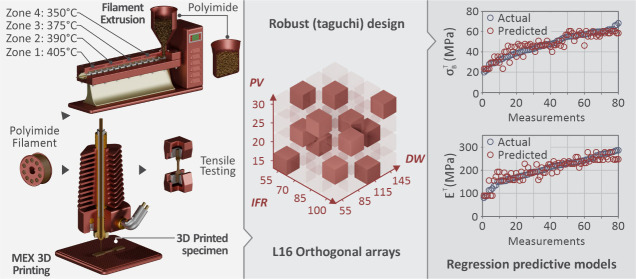

## 1 Introduction

There is a great variety of polymeric materials that can be categorized as commodity, engineering, or high-performance.^1^ High-performance polymers (HPPs) are characterized by their high temperature, chemical resistance, and strength.^2^ Existing HPPs include polyether imide (PEI),^3^ polyether ether ketone (PEEK),^4^ polyetherketoneketone (PEKK),^5^ polyvinylidene fluoride (PVDF),^6^ poly­(phenylene sulfide) (PPS),^7^ poly­(poly­(ether sulfone)) (PES),^8^ polyphenylenesulfone (PPSU),^9^ polysulfone (PSU),^10^ and polyimide (PI).^11^

HPPs are becoming increasingly popular because of their superior thermomechanical properties.^12^ The most remarkable advantage of these materials is their ability to withstand extreme conditions and maintain their properties. Such features make them preferable for a wide variety of demanding applications.^13^ They have been utilized in the medical field,^14^ especially in dentistry^15,16^ and orthopedical,^17,18^ in aerospace,^19,20^ electrical,^21,22^ and other emerging applications such as oil and gas, textiles, and solar energy.^23–25^

The great interest in the utilization of HPPs inevitably leads to a gradual increase in their market size.^26–28^ According to a report by Grand View Research,^29^ in 2024, the global HPPs market size was calculated to be approximately USD 26,750.06 million. It is expected to reach a compound annual growth rate (CAGR) of 9.32% between 2025 and 2030.

PI is considered an organic polymeric material with repeating units as part of the main chain, which contains an imide group (−CO-NH–CO−).^30^ It is high- and low-temperature resistant,^31^ and possesses excellent mechanical^32^ and dielectric properties,^33,34^ low moisture absorption, and chemical and radiation resistance.^11,35^ It is characterized by its low weight, flexibility, flame retardancy, and physical and chemical properties.^36^ PI can be found in various forms, such as films,^37^ coatings,^38^ varnishes,^39^ binders,^40^ tapes,^41^ fibers,^42^ composites,^43^ foams.^44^

It is an excellent dielectric material^45^ for electric energy storage,^46,47^ electronics,^48,49^ photoresists,^50^ skin-inspired electronics,^51^ and many more applications.^52^ It is also worth mentioning that PI has been applied in the healthcare sector,^53,54^ as an antibacterial material,^55^ for drug delivery,^56–59^ biosensors,^60–62^ tissue replacement,^63–66^ respirators,^67,68^ etc. Moreover, PI can be useful for military armor, aerospace and aviation parts, microelectronics, solar-to-electrochemical energy storage, photo- or electro-catalysis, etc.^69–71^ The polyimide market size, as indicated by Research and Markets, is expected to have a 9.0% CAGR between 2025 and 2030, reaching USD 1.83 billion by the year 2030.^72^

AM has made progress in employing HPPs^73^ for various applications in different fields. This is a significant development, as it can provide alternatives to several issues concerning the scientific and industrial sectors. Furthermore, optimization efforts have been made for the manufacturing, properties, and performance of the produced parts. Some of the existing optimization designs include the full factorial design,^74,75^ Taguchi design,^76–78^ Box-Behnken design,^79–81^ Doehlert experimental design,^82^ definitive screening design,^83^ and Placket-Burmann design.^84^ As the literature review revealed, these are commonly used methods for optimizing 3D printed parts made with engineering^85–90^ and high-performance polymers.^91,92^ They have proven their efficacy in different types of research content related to bioplotting, hybrid AM, mechanical performance, quality metrics of 3D printed parts, etc.^93–96^

Several optimization efforts have been reported in research related to HPPs’ behavior,^97,98^ such as the Taguchi method on PEEK,^92,99,100^ PEI,^101,102^ and Box-Behnken method on PEEK,^80,103^ aiming to improve their mechanical performance or quality characteristics. However, the range of research on PI 3D printing and the optimization process needs to be expanded to enrich the existing literature and related knowledge on this issue.^104^

To the best of the authors’ knowledge, no integrated research work has been conducted on the optimization of PI 3D printing parameters to enhance mechanical metrics. The key innovation of this research is that it is the first to follow a systematic statistical optimization model for polyimide (PI) in material extrusion (MEX) 3D printing. Polyimide literature is focused on extrusion-based additive manufacturing. There has been little investigation into optimizing this process. By combining the Taguchi method, analysis of variance (ANOVA), and regression modeling, this study shifts the research from experimental trial-and-error efforts to a robust and quantifiable approach. Findings contribute to the prediction and optimization of the mechanical response of the PI MEX 3D printed components. This method provides the effect of the process factors on the mechanical performance of the MEX 3D printed PI parts. The process factor interactions are reported as well.

Optimizing the 3D printing parameters for high-performance PI is critical for exploiting the superior thermal, chemical, and mechanical properties of the material. The performance of the PI is sensitive to the process parameters. The systematic approach followed (Taguchi design of experiment, DOE in combination with regression analysis) enabled the production of reproducible and high-quality parts. It also reduces material waste and costs, which is critical considering the high cost of high-performance materials such as PI. Moreover, this is critical as high-performance polymers such as PI are utilized in demanding applications and hazardous environments because of their advanced characteristics.

In this study, five 3D printing settings were employed: raster orientation (RO), hot-end temperature (HT), printhead velocity (PV), internal fill ratio (IFR), and deposition width (DW). They were applied at different levels to determine the optimum set of parameters, achieving the highest performance in uniaxial loading scenarios (tensile testing). The chosen response parameters were tensile strength (σ_*B*_^*T*^), tensile yield strength (σ_*Y*_^*T*^), Young’s modulus (*E* ^*T*^), and tensile toughness (*T* ^*T*^).

They were studied using a Taguchi L16 design. Two regression approaches were applied: one with a Reduced Quadratic Regression Model (RQRM) and the other with a Linear Regression Model (LRM). The RQRM was proven to be more effective for the needs of this research and was, thus, selected for utilization. Sixteen (16) experimental runs were conducted along with two (2) additional confirmation runs. Confirmation runs provided correlation data in relation to the prediction models compiled through regression analysis.

The merit of such a systematic optimization effort is high compared to the existing literature works, which focus solely on experimental processes. The research on the high-performance PI in the MEX AM is still limited. The reasons can be assumed to be related to the high cost of the high-performance polymers, the special equipment required for their processing (filament extrusion and 3D printing), and the difficulties in their processing compared to commodity thermoplastics. For these reasons, optimization of the performance of parts made with the PI high-performance polymer with the MEX AM is of high importance. Especially considering also that the high-performance polymers operate in demanding environments in terms of mechanical loading, thermal and chemical exposure, etc., due to their specs. Herein, the optimum set of parameters is reported, along with the 3D printing parameters that affect the most each tensile test property considered in the research. Furthermore, the prediction formulas compiled were verified and could have direct industrial use. This allows for more accurate prediction than either empirical or trial and error approaches, therefore, providing a more scientific implementation for the utilization of PI polymers in MEX AM.

Part of this work also examined the thermal PI characteristics through thermogravimetric analysis (TGA) and differential scanning calorimetry (DSC) for completeness and to document the temperature levels used in the study. Surface microscopy was conducted by using scanning electron microscopy (SEM) on the lateral and fractured surfaces of the specimens. The information extracted from this research can be valuable for expanding the PI 3D printing-related literature; thus, creating opportunities for new applications, further exploiting the unique features of high-performance polymers, such as PI.

## 2 Materials and Methods

### 2.1 Experimental Procedure

PI (also known as TPI, i.e., thermoplastic PI) pellets of AURUM PL450C grade were supplied by Mitsui Chemicals (Tokyo, Japan). Based on the information stated by the supplier in the related datasheet, its characteristics are specific gravity 1.33, elongation 90% (ASTM D638), tensile strength 92 MPa (ASTM D638), Izod impact strength 88J/m (ASTM D256), flexural strength 137 MPa (ASTM D790), Rockwell hardness (R scale) 129 (ASTM D785), and heat distortion temperature 230 °C (ASTM D648).

The experimental procedure of this research is described by the images and their related captions in Figure 1 (left). The right part of Figure 1 presents the experimental modeling strategy in flowchart form, including the variable control parameters, output metrics, and fixed control parameters. Initially, material oven drying (Figure 1a), filament extrusion (Figure 1b), and filament drying (Figure 1c) were performed, followed by MEX 3D printing (Figure 1d), dimensional evaluation (Figure 1e), quality control (Figure 1f), and optical microscopy (Figure 1g).

**Figure 1:**
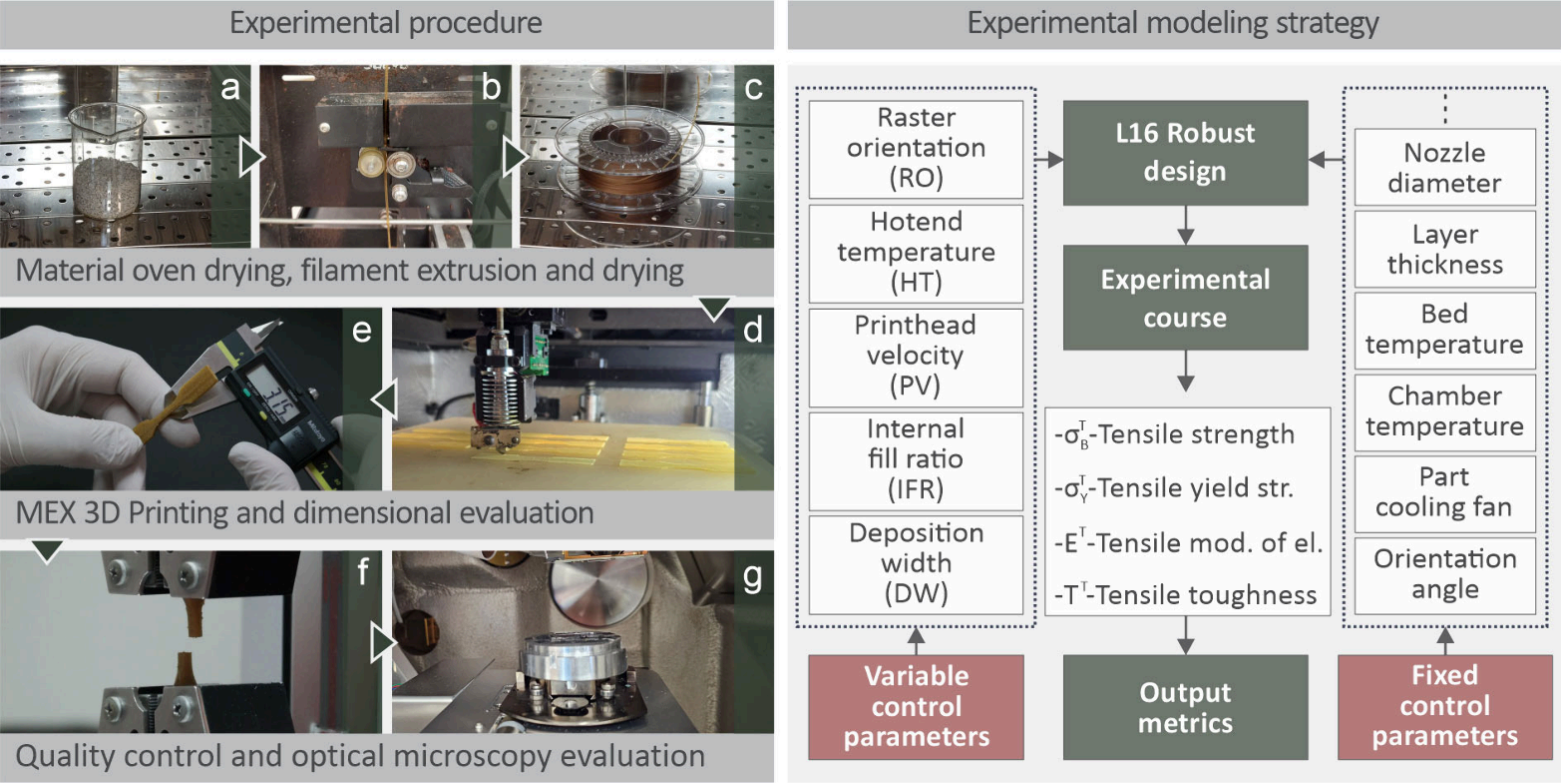
(Left side) Followed experimental procedure (a) drying of the raw material, (b) extrusion of raw material to filament, (c) drying of the produced filament, (d) specimen 3D printing, (e) dimensional inspection, (f) quality control, and (g) optical microscopy.

### 2.2 TGA/DSC

Thermal evaluation of the PI material was conducted through thermogravimetric analysis (TGA) and differential scanning calorimetry (DSC), with the aim of examining the effect of temperature applied during the research on the degradation of the material. A PerkinElmer Diamond (Waltham, Massachusetts, U.S.) was used to obtain the TGA results, and a TA Instruments Discovery-Series DSC 25 (from Delaware, U.S.) was used for the DSC. The results are shown in Figure 2. The TGA curves are shown in the top right section of Figure 2a. Their derivatives are shown in the bottom section. The DSC endothermic/heating curves are presented in the top part of Figure 2b and their derivatives are presented in the bottom section, whereas the respective exothermic/cooling DSC curves are shown in Figure 2c.

**Figure 2:**
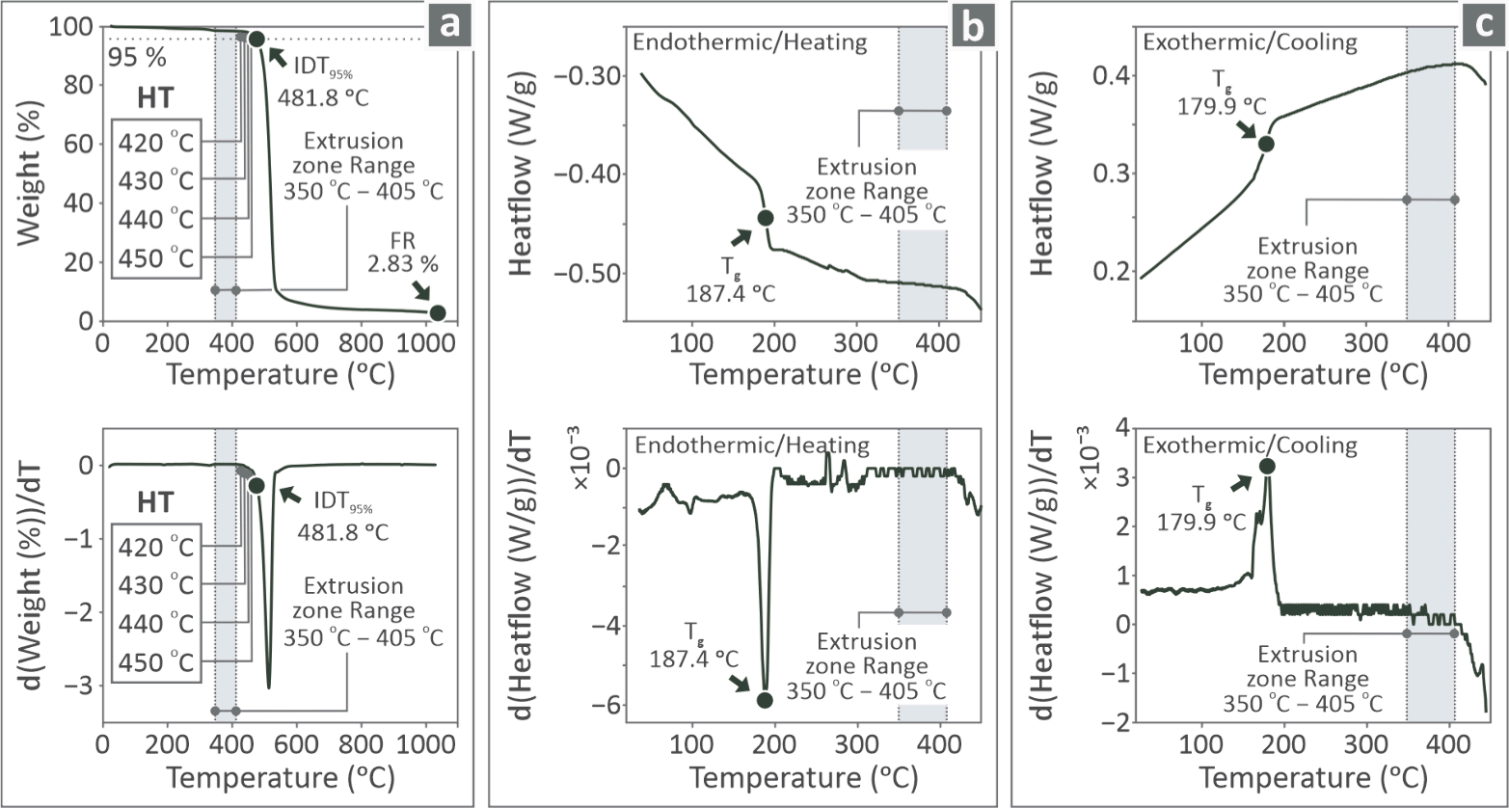
(a) TGA curve at the top section and its derivative at the bottom section, 3D printing temperatures utilized, (b) DSC endothermic/heating curve at the top section and its derivative at the bottom section, and (c) the respective information about the DSC exothermic/cooling curve.

### 2.3 Filament Extrusion and Specimen 3D Printing

Filament extrusion was conducted using a 3devo Precision 450 extruder (Utrecht, The Netherlands) set to provide 350 °C in zone 4 (hopper), 375 °C in zone 3, 390 °C in zone 2, and 405 °C in zone 1 (nozzle), 7 rpm screw speed, and 30% cooling fan speed. Filament production was followed by drying in a laboratory oven at 120 °C for 8 h, as suggested by the standard recommendations for polyimide-based materials. The filament was then supplied to an Intamsys Funmat HT (the company is established in Shanghai, China) printer to manufacture the coupons. The design, dimensions, and standards used for manufacturing the tensile specimens are shown in Figure 3c. The fixed and variable control parameters set during the 3D printing of the PI are presented in Figure 3b. The fixed parameters were layer thickness: 0.2 mm, nozzle diameter: 0.4 mm, bed temperature: 160 °C, chamber temperature: 90 °C, part cooling fan: 0 (disabled) %, orientation angle: 0 deg, infill pattern: linear, number of perimeters: 2. As far as it concerns the variables, raster orientation (RO) varied between 0, 30, 60, and 90 deg; hot-end temperature (HT) between 420, 430, 440, and 450 °C; printhead velocity (PV) between 15, 20, 25, and 30 mm/s; internal fill ratio (IFR) between 55, 70, 85, and 100%; deposition width (DW) between 55, 85, 115 and 145%. Figure 3 also shows a schematic of the Taguchi design: L16 orthogonal arrays. The Taguchi L16 model under examination consists of five control parameters with four levels each. The cube had three directions, meaning that only three parameters could be observed. Therefore, the three most important ones were selected, as shown in Figure 3 (Ranks 1, 2, and 3), based on their effect σ_*B*_^*T*^ and σ_*Y*_^*T*^.

**Figure 3:**
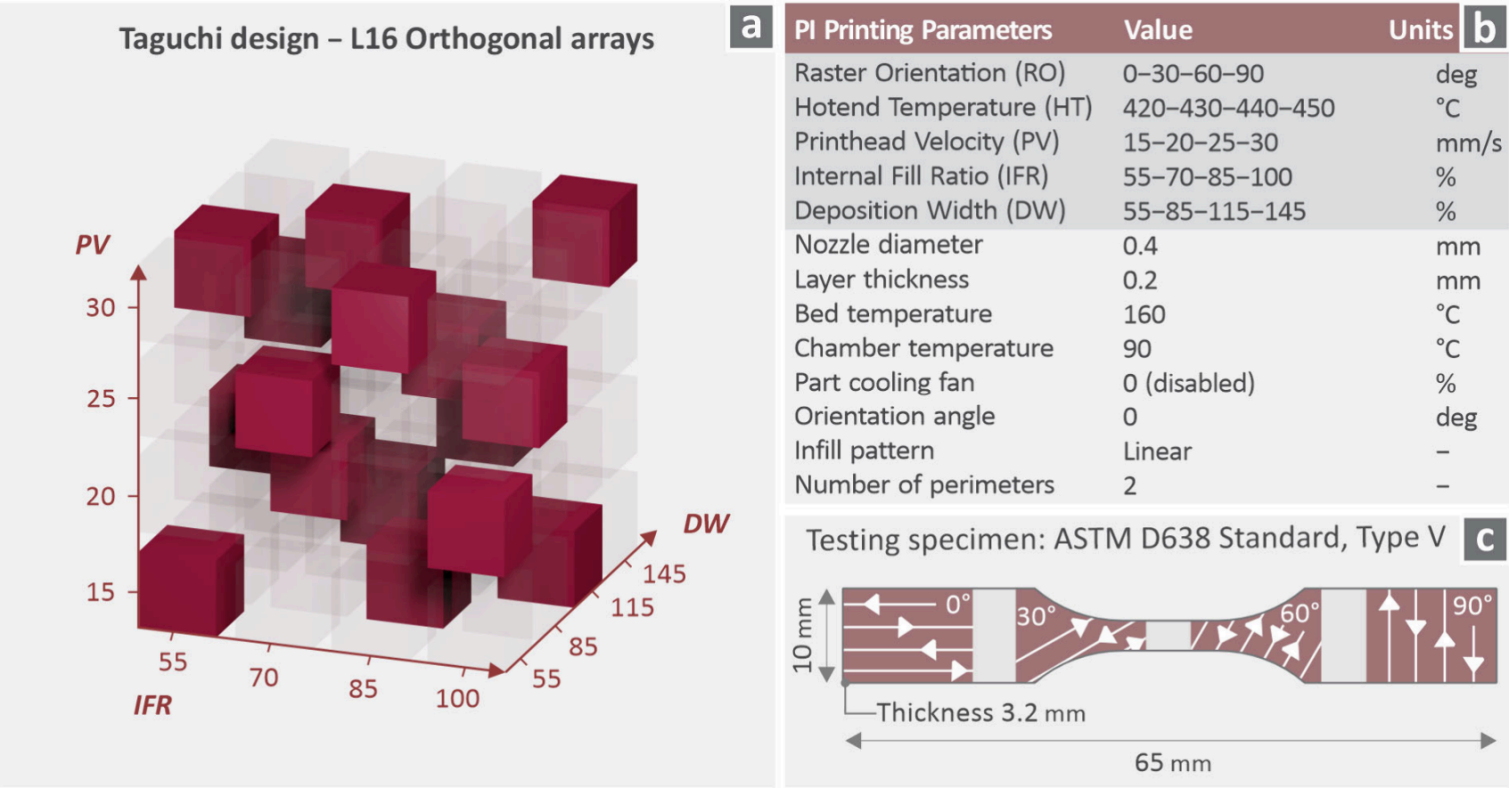
(a) Illustration of Taguchi design: L16 orthogonal arrays, (b) PI fixed and variable parameters, (c) testing specimen design, dimensions, and followed ASTM (D638).

### 2.4 Tensile Testing and Microscopy Evaluation

The specimens were tensile tested using an Imada MX2 (from Northbrook, Illinois, United States) (V-type, 3.2 mm thick). For the morphological examination of the produced and tested tensile specimens, their lateral and fractured surfaces were subjected to SEM, and images were captured at various magnifications. These were obtained using a JSM 6362LV (field emission apparatus from Jeol Ltd., Peabody, Massachusetts, U.S., Au sputtered samples, high vacuum, 20 kV).

### 2.5 Taguchi L16, ANOVA, Regression

The wide range of tests and necessary manufactured specimens that are required when following a classical experimental design method create the need for utilizing less complex designs. Thus, an increase in the number of parameters selected for examination can make the processing even more difficult. Consequently, the Taguchi design of experiment (DOE) was utilized for modeling by forming an orthogonal array. It was then used to examine the influence of the different modeled parameters assessed on the responses, and to discover the most beneficial combination of parameters.^105^ Taguchi found applications in various research fields,^84^ as it has proven successful.^106–110^ The special design of the orthogonal arrays utilized by the Taguchi method examines the entire parameter space, resulting in the need for limited experiments to solve this problem.^111^ Orthogonal array selection is typically performed using the total degrees of freedom (DOF). Every factor possesses a DOF that can be found by subtracting one from the number of factor levels.^112–117^ This is presented more analytically in the Supporting Information section.

The chosen Taguchi L16 included 16 (16) experiments with five (5) repetitions each, resulting in 80 (80) response sets. Five (5) control parameters were employed (RO, °, HT, °C, PV, mm/s, IFR, %, and DW, %) and are listed in Table 1, along with their levels. Their levels were determined by consulting the material datasheet (for HT) and evaluating the PI printability in preliminary tests (for the remaining control parameters).

**Table 1:** Taguchi L16 Design: Control Parameters and Levels

**Run**	**RO (deg)**	**HT (°C)**	**PV** **(mm/s)**	**IFR (%)**	**DW (%)**
1	0	420	15	55	55
2	0	430	20	70	85
3	0	440	25	85	115
4	0	450	30	100	145
5	30	420	20	85	145
6	30	430	15	100	115
7	30	440	30	55	85
8	30	450	25	70	55
9	60	420	25	100	85
10	60	430	30	85	55
11	60	440	15	70	145
12	60	450	20	55	115
13	90	420	30	70	115
14	90	430	25	55	145
15	90	440	20	100	55
16	90	450	15	85	85

The statistical technique of ANOVA is useful for interpreting experimental results by determining the contribution ratio of each parameter. The importance of each parameter concerning the solution to the problem was also determined using ANOVA. The calculation steps are provided in the Supplementary File.^118,119^ A prediction model for the output metrics as an equation for the control parameters was developed by using regression methods. Corresponding equations were created for every regression method to compare their accuracies and determine the possibility of utilizing simpler equations for modeling. Critical statistical parameters for the evaluation of the analysis were calculated and are provided. R^2^ is the coefficient of determination. It is a statistical measure that shows the proportion of the variance in the dependent variable (the response) that is explained by the independent variables (the predictors) in a regression model. R^2^(pred), also called the predictive R-squared, is a statistic that measures how well a regression model predicts new data. R^2^(adj) is the adjusted R^2^. It is a modified version of the coefficient of determination that takes into account the number of predictors in the model relative to the number of data points. The normal R^2^ always increases when adding more predictors, even if those predictors do not really improve the model. R^2^(adj) increases only when a new term genuinely improves the model more than would be expected. Furthermore, two additional runs were performed as confirmation runs to assess the precision of the predictive models.

## 3 Results

### 3.1 Tensile Test and SEM Results

In Figure 4, four cases of experiments are presented, as they belong to the diagonal of the total number of runs: Run 1 (Figure 4a), Run 6 (Figure 4b), Run 11 (Figure 4c), and Run 16 (Figure 4d) constitute the diagonal. For a specific randomly selected replica of each run, the respective variable control parameters are depicted, while the tensile curves indicate the σ_*B*_^*T*^, σ_*Y*_^*T*^ and *E* ^*T*^, as well as an SEM image captured from the fractured surface at 5000×. It is remarkable that both σ_*B*_^*T*^ and σ_*Y*_^*T*^ of Run 6 presented the highest levels, whereas the lowest levels were detected in the case of Run 11. The brittle behavior appears to characterize all of the run samples by examining the SEM images.

**Figure 4:**
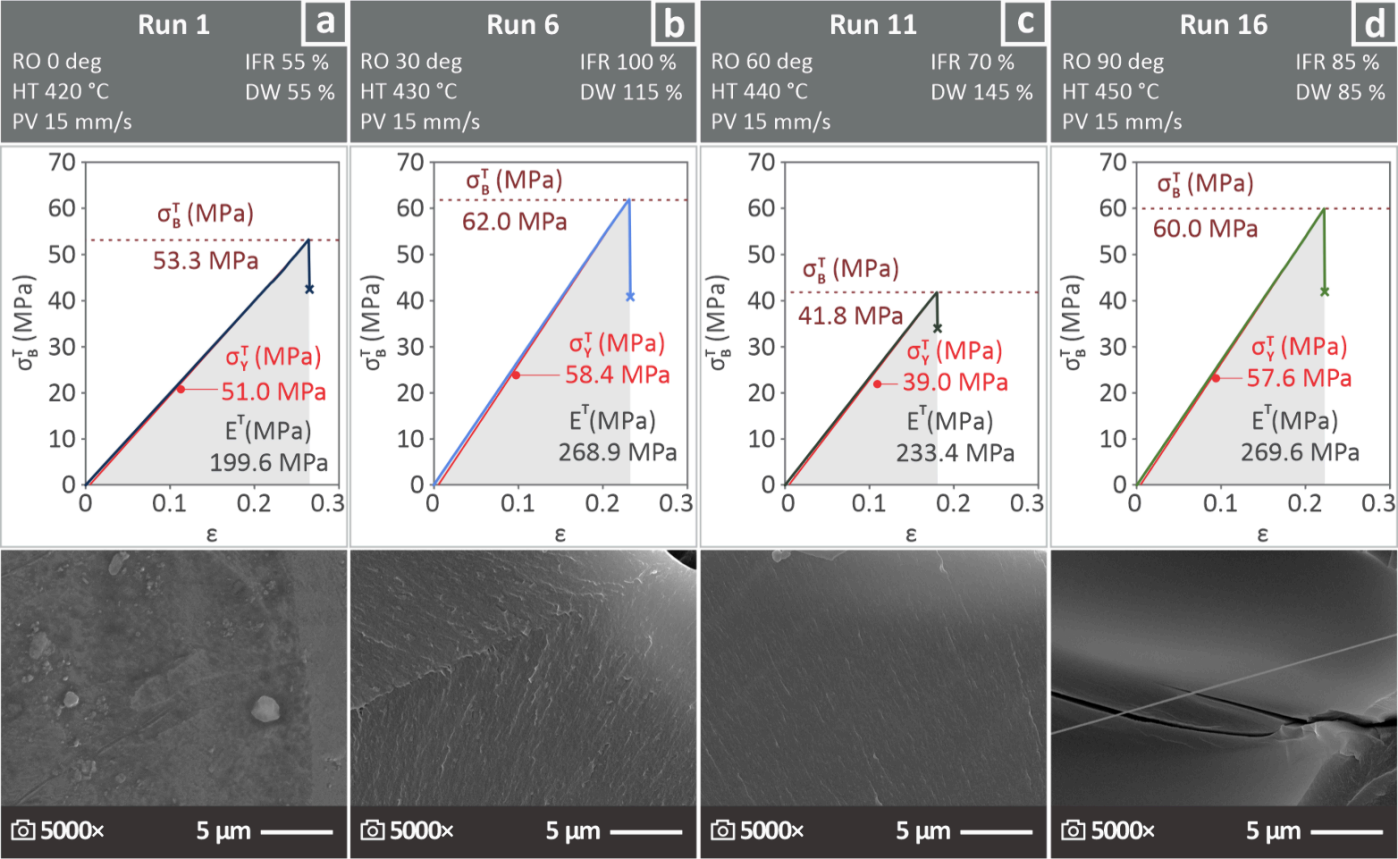
Variable control parameters, the tensile curves reveal σ_*B*_^*T*^, σ_*B*_^*T*^ and *E* ^*T*^, and SEM pictures of fractured surfaces belonging to Run (a) 1, (b) 6, (d) 11, and (d) 16 of the formed diagonal array.

Figure 5 shows SEM images (at 27× magnification) from randomly selected specimens belonging to all runs examined (1–16, Figure 5a–p respectively), indicating their fractured surfaces as a result of tensile testing. The diagonal in Figure 4 is outlined in red (runs 1, 6, 11, and 16). The specimens appear to have pores and some voids, while some of them seem to exhibit brittle behavior and others exhibit ductile behavior owing to the applied 3D printing parameter set.

**Figure 5:**
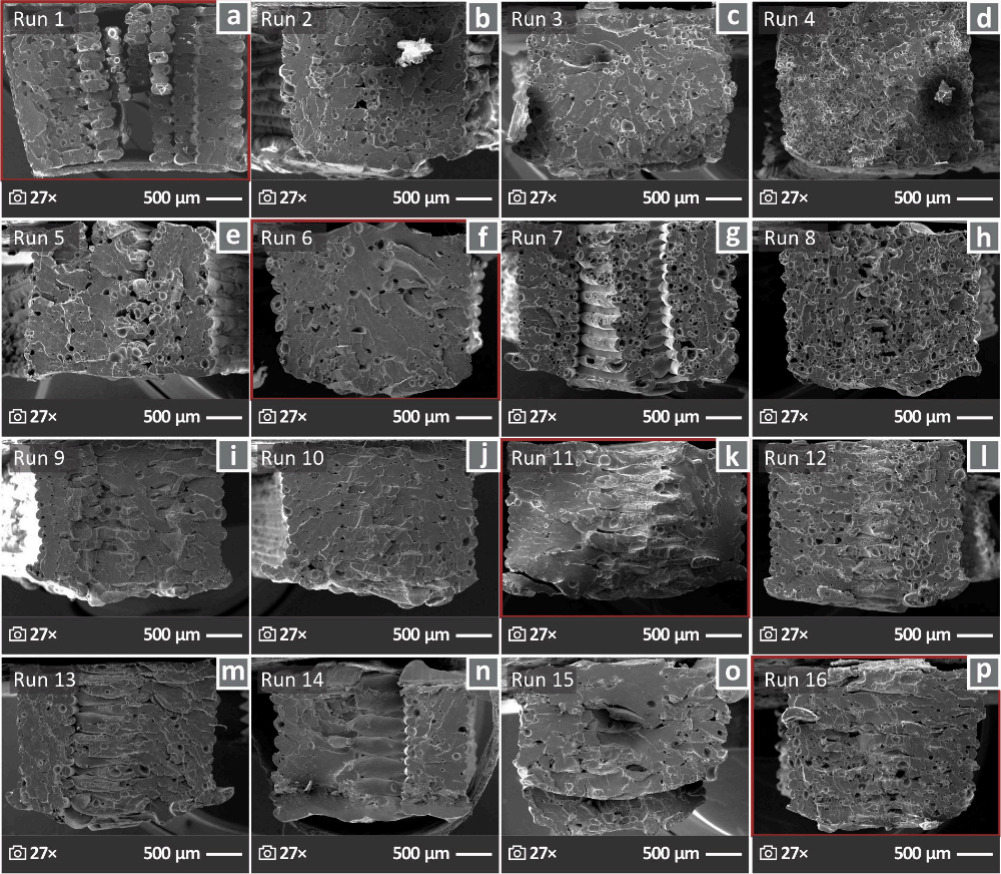
Fractured surfaces of samples in SEM images of 27× magnification for (a-p) 1–16 runs, and the diagonal is highlighted with a red outline.

Figure 6 shows the SEM images of the samples from the diagonal, namely, Run 1 (Figure 6a,e,i), Run 6 (Figure 6b,f,j), Run 11 (Figure 6c,g,k), and Run 16 (Figure 6d, h, l). Figure 6a–d presents the lateral surfaces magnified 27× while Figure 6e–h and i–l show the fractured surfaces at 300 and 40,000×, respectively.

**Figure 6:**
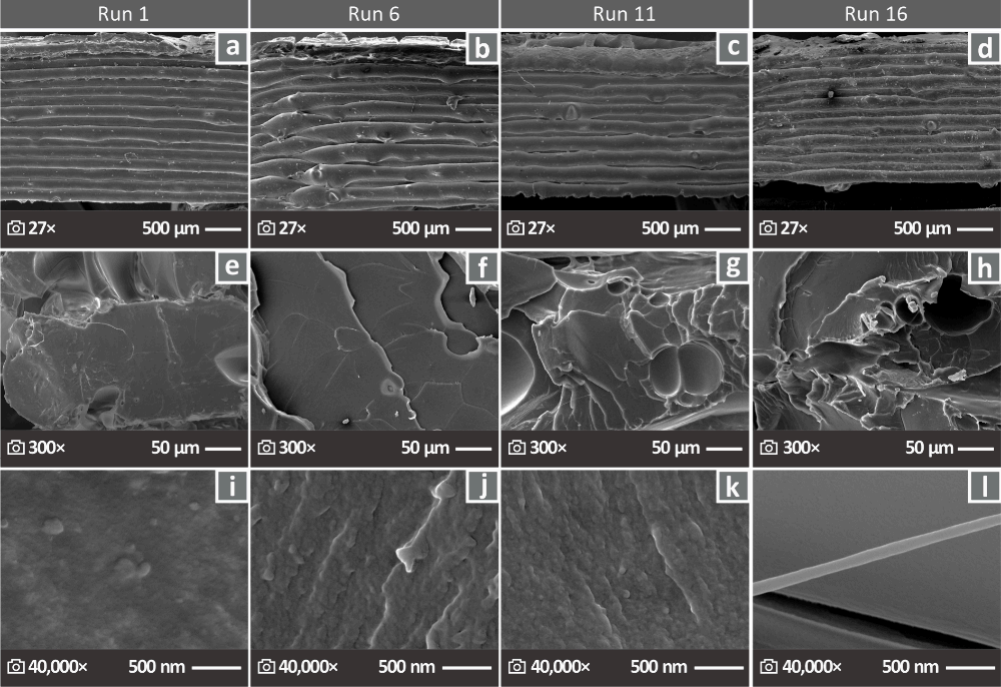
SEM images with regard to the diagonal derived Run 1, Run 6, Run 11, and Run 16, (a-d) lateral surfaces in 27× magnification, (e-h) fractured surfaces in 300×, and (i-l) 40,000× magnification

### 3.2 Experimentally Derived Results

Table 2 contains the RO, HT, PV, IFR, and DW control parameters’ ranking for σ_*B*_^*T*^, σ_*B*_^*Y*^, *E* ^*T*^ and *T* ^*T*^ responses, (considering delta statistics). Each factor was calculated by subtracting the lowest average value from the highest. The greatest and least significant effects on the response metric were detected through the highest and lowest delta values, which were placed in ranks 1 and 5, respectively. As shown in Table 2, PV was ranked 1 in all cases except for *T* ^*T*^ which had IFR, and HT was ranked 5 in all cases except for *E* ^*T*^ which had RO. The four levels of each control parameter and their average values are listed in Table 2. Table 3 provides the average and standard deviation values for σ_*B*_^*T*^, σ_*B*_^*Y*^, *E* ^*T*^ and *T* ^*T*^ (with regard to the 16 (16) runs). The standard deviation was calculated from the five repetitions of each run for each response metric. Table S1, included in the Supporting Information, possesses additional experimental data for the 16 (16) experimental runs and all of their replicas.

**Table 2:** Control Parameters Ranking for Means σ_*B*_^*T*^, σ_*Y*_^*T*^, *E* ^*T*^, *T* ^*T*^

**Level**	**RO (deg)**	**HT (°C)**	**PV** **(mm/s)**	**IFR (%)**	**DW (%)**
**σ**_***B***_^***T***^ **(** ***MPa*** **)**					
**1**	53.02	41.68	53.18	40.58	52.21
**2**	50.94	48.33	53.16	46.18	48.58
**3**	43.04	48.89	48.06	50.56	48.3
**4**	43.23	51.34	35.82	52.91	41.15
**Delta**	9.99	9.66	17.36	12.33	11.06
**Rank**	4	5	1	2	3
**σ**_*Y*_^***T***^ **(** ***MPa*** **)**					
**1**	50.54	38.82	50.23	38.54	49.56
**2**	48.32	45.82	50.05	43.92	45.56
**3**	40.1	46.28	45.41	47.91	45.86
**4**	40.96	49	34.22	49.55	38.93
**Delta**	10.44	10.18	16.01	11.01	10.62
**Rank**	4	5	1	2	3
***E*** ^***T***^ **(** ***MPa*** **)**					
**1**	215.8	168.4	239.2	185.5	229.3
**2**	216.8	209.9	223.1	207.9	204.3
**3**	196.8	217	216.9	206.8	203.3
**4**	198.1	232.2	148.2	227.2	190.5
**Delta**	20	63.8	91	41.7	38.7
**Rank**	5	2	1	3	4
***T*** ^***T***^ **(** ***MJ/m*** ^**3**^ **)**					
**1**	6.715	5.629	6.344	4.79	6.402
**2**	6.362	5.948	6.392	5.814	6.163
**3**	5.297	5.882	5.969	6.55	6.173
**4**	5.369	6.285	5.038	6.59	5.004
**Delta**	1.418	0.656	1.354	1.8	1.398
**Rank**	2	5	4	1	3

**Table 3:** Average and Standard Deviations Values of measured Responses for σ_*B*_^*T*^, σ_*Y*_^*T*^, *E* ^*T*^, *T* ^*T*^

**Run**	**σ**_***B***_^***T***^ **(** **MPa** **)**	**σ**_*Y*_^***T***^ **(** **MPa** **)**	***E*** ^***T***^ **(** **MPa** **)**	***T*** ^***T***^ **(** **MJ** **/** **m** ^**3**^ **)**
1	50.44 ± 4.30	47.77 ± 4.54	210.72 ± 22.01	6.14 ± 0.58
2	59.04 ± 4.19	55.98 ± 4.57	233.51 ± 12.78	7.29 ± 0.60
3	58.60 ± 2.57	56.09 ± 2.96	232.45 ± 11.02	7.55 ± 0.48
4	44.02 ± 6.96	42.32 ± 7.62	186.39 ± 20.52	5.89 ± 1.08
5	47.26 ± 7.68	44.12 ± 8.03	178.22 ± 30.13	6.19 ± 1.13
6	63.44 ± 3.84	59.87 ± 4.12	268.91 ± 9.88	7.67 ± 0.71
7	34.58 ± 5.08	33.02 ± 5.61	144.44 ± 22.50	4.49 ± 0.78
8	58.50 ± 1.71	56.29 ± 1.91	275.55 ± 14.02	7.09 ± 0.35
9	44.03 ± 4.76	39.53 ± 3.96	186.21 ± 21.18	5.90 ± 0.79
10	39.72 ± 5.91	37.68 ± 6.24	163.49 ± 22.45	5.49 ± 1.05
11	42.20 ± 4.30	39.54 ± 4.49	223.98 ± 24.83	4.60 ± 0.41
12	46.19 ± 2.87	43.63 ± 3.21	213.54 ± 12.89	5.19 ± 0.56
13	24.97 ± 5.53	23.86 ± 5.92	98.48 ± 18.06	4.28 ± 0.94
14	31.12 ± 3.18	29.75 ± 3.50	173.49 ± 12.93	3.34 ± 0.48
15	60.17 ± 2.86	56.48 ± 3.27	267.28 ± 12.76	6.89 ± 0.39
16	56.65 ± 2.14	53.74 ± 2.44	253.18 ± 18.18	6.97 ± 0.30

### 3.3 ANOVA Data and Regression Equations

Figure 7 indicates the effect of each parameter on all of the responses under investigation, while the ranks are also included, showing the importance of each parameter based on their influence on the responses. Figure 7a shows σ_*B*_^*T*^ (dark colored) and σ_*Y*_^*T*^ (red colored), whereas Figure 7b shows *E* ^*T*^ (dark colored) and *T* ^*T*^ (red colored). PV appears to be the parameter ranked first when it comes to all responses, except for *T* ^*T*^, where the IFR had the greatest impact. In contrast, HT was ranked fifth in all cases except for *E* ^*T*^ where RO was the least influential. The best values representing the highest desired levels are highlighted with a box placed around them, as listed in Table 2.

**Figure 7:**
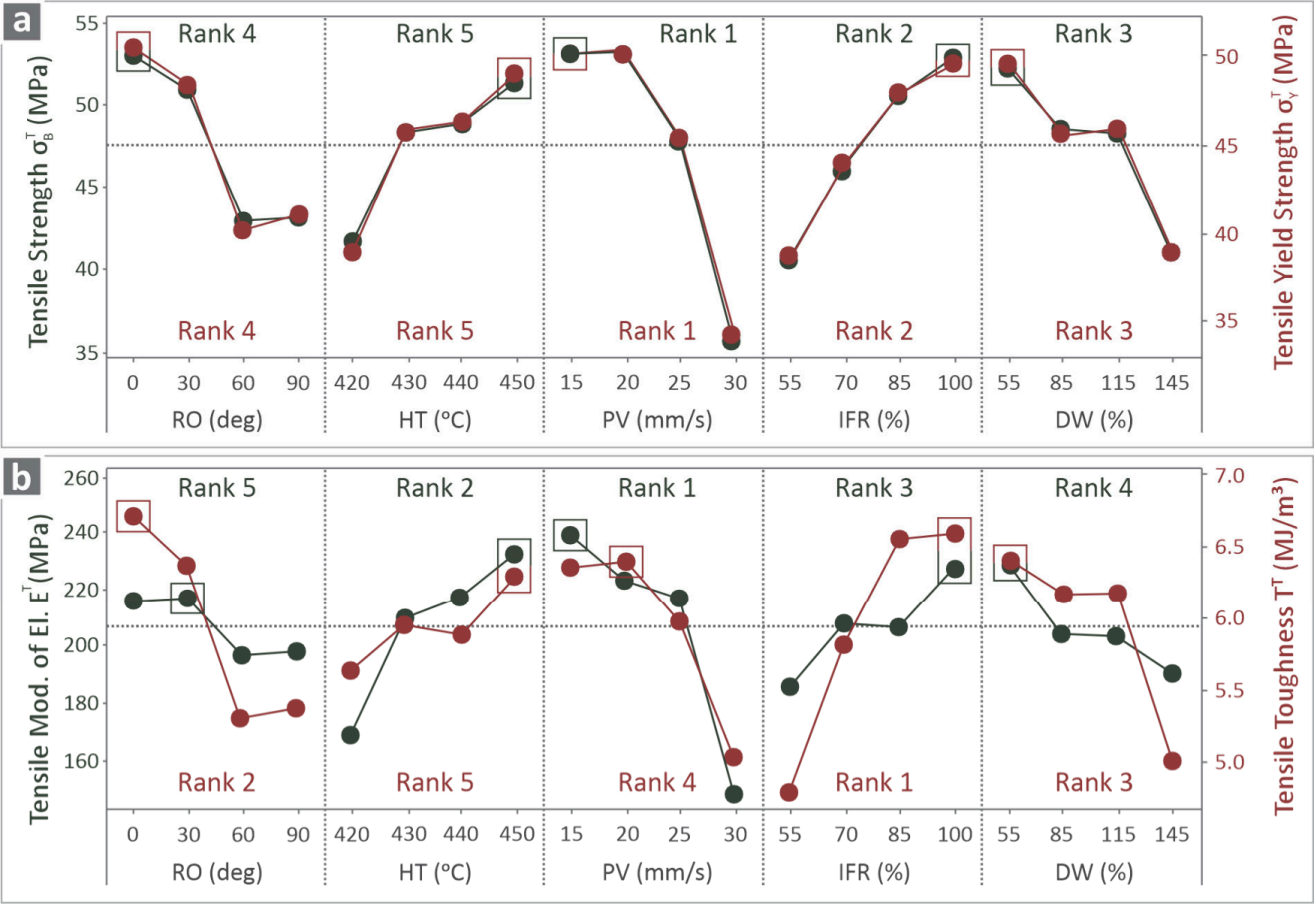
Main effect plots of (a) σ_*B*_^*T*^ (dark colored) and σ_*Y*_^*T*^ (red colored), (b) *E* ^*T*^ (dark colored) and *T* ^*T*^ (red colored) vs the RO, HT, PV, IFR, and DW responses, and their ranks.

RQRM was the selected regression model in order to obtain the desired results, which are presented in the next tables for the four response metrics, i.e., σ_*B*_^*T*^ (Table 4), σ_*B*_^*Y*^ (Table 5), *E* ^*T*^ (Table 6), and *T* ^*T*^ (Table 7) vs RO, HT, PV, IFR, and DW. Each table is accompanied by the respective equations used for the calculation of every response metric (eqs 1–4).

**Table 4:** Polynomial ANOVA, Reduced Quadratic Regression Model σ_B_^T^ vs RO, HT, PV, IFR, DW

Source	DF	Adj SS	Adj MS	F-Value	P-Value
Regression	10	9339.16	933.916	38.07	0.000
*RO*	1	241.24	241.242	9.83	0.003
*HT*	1	94.03	94.033	3.83	0.054
*PV*	1	464.14	464.145	18.92	0.000
*IFR*	1	116.17	116.169	4.74	0.033
*DW*	1	11.26	11.259	0.46	0.500
*RO* ^2^	1	25.90	25.901	1.06	0.308
*HT* ^2^	1	88.24	88.243	3.60	0.062
*PV* ^2^	1	747.06	747.059	30.45	0.000
*IFR* ^2^	1	52.37	52.373	2.13	0.149
*DW* ^2^	1	61.97	61.974	2.53	0.117
Error	69	1692.69	24.532		
Total	79				
R^2^	84.66%				
R^2^ (adj)	82.43%				
R^2^ (pred)	79.27%				

**Table 5:** Polynomial ANOVA, Reduced Quadratic Regression Model σ_*Y*_^*T*^ vs RO, HT, PV, IFR, and DW

Source	DF	Adj SS	Adj MS	F-Value	P-Value
Regression	10	8326.28	832.628	29.63	0.000
*RO*	1	294.05	294.053	10.46	0.002
*HT*	1	98.05	98.046	3.49	0.066
*PV*	1	372.91	372.912	13.27	0.001
*IFR*	1	132.46	132.455	4.71	0.033
*DW*	1	5.34	5.337	0.19	0.664
*RO* ^2^	1	47.24	47.235	1.68	0.199
*HT* ^2^	1	91.85	91.848	3.27	0.075
*PV* ^2^	1	607.15	607.150	21.61	0.000
*IFR* ^2^	1	69.69	69.694	2.48	0.120
*DW* ^2^	1	43.11	43.109	1.53	0.220
Error	69	1938.90	28.100		
Total	79				
R^2^	81.11%				
R^2^ (adj)	78.37%				
R^2^ (pred)	74.46%				

**Table 6:** Polynomial ANOVA, Reduced Quadratic Regression Model, *E* ^*T*^ vs RO, HT, PV, IFR, and DW

Source	DF	Adj SS	Adj MS	F-Value	P-Value
Regression	10	169843	16984.3	33.89	0.000
*RO*	1	461	461.1	0.92	0.341
*HT*	1	3707	3706.8	7.40	0.008
*PV*	1	8023	8022.6	16.01	0.000
*IFR*	1	227	227.0	0.45	0.503
*DW*	1	1798	1797.9	3.59	0.062
*RO* ^2^	1	0	0.4	0.00	0.977
*HT* ^2^	1	3463	3462.9	6.91	0.011
*PV* ^2^	1	13867	13867.2	27.67	0.000
*IFR* ^2^	1	19	19.3	0.04	0.845
*DW* ^2^	1	732	732.0	1.46	0.231
Error	69	34575	501.1		
Total	79				
R^2^	83.09%				
R^2^ (adj)	80.63%				
R^2^ (pred)	77.27%				

**Table 7:** Polynomial ANOVA, Reduced Quadratic Regression Model, *T* ^*T*^ vs RO, HT, PV, IFR, DW

Source	DF	Adj SS	Adj MS	F-Value	P-Value
Regression	10	118.546	11.8546	20.93	0.000
*RO*	1	5.619	5.6191	9.92	0.002
*HT*	1	0.029	0.0288	0.05	0.822
*PV*	1	3.054	3.0542	5.39	0.023
*IFR*	1	7.405	7.4049	13.08	0.001
*DW*	1	2.266	2.2659	4.00	0.049
*RO* ^2^	1	0.907	0.9073	1.60	0.210
*HT* ^2^	1	0.036	0.0358	0.06	0.802
*PV* ^2^	1	4.786	4.7860	8.45	0.005
*IFR* ^2^	1	4.841	4.8408	8.55	0.005
*DW* ^2^	1	4.326	4.3262	7.64	0.007
Error	69	39.077	0.5663		
Total	79				
R^2^	75.21%				
R^2^ (adj)	71.62%				
R^2^ (pred)	66.52%

1 $$\sigma_{B}^{T}=-2133-0.1812\times RO+9.43\times HT+4.36\times PV+0.833\times IFR+0.084\times DW+0.000632\times RO^{2}-0.01050\times HT^{2}-0.1222\times PV^{2}-0.00360\times IFR^{2}-0.000978\times DW^{2}$$

2 $$\sigma_{Y}^{T}=-2178-0.2001\times RO+9.63\times HT+3.91\times PV+0.890\times IFR+0.058\times DW+0.000854\times RO^{2}-0.01071\times HT^{2}-0.1102\times PV^{2}-0.00415\times IFR^{2}-0.000816\times DW^{2}$$

3 $$E^{T}=-13219-0.251\times RO+59.2\times HT+18.11\times PV+1.16\times IFR-1.063\times DW+0.00008\times RO^{2}-0.0658\times HT^{2}-0.527\times PV^{2}-0.0022\times IFR^{2}+0.00336\times DW^{2}$$

4 $$T^{T}=25-0.02766\times RO-0.165\times HT+0.353\times PV+0.2104\times IFR+0.0377\times DW+0.0001183\times RO^{2}+0.000211\times HT^{2}-0.00978\times PV^{2}-0.001093\times IFR^{2}-0.0002584\times DW^{2}$$

Table 8 presents the results derived after comparing the two regression models of LRM and RQRM, based on which the proper model for servicing this investigation was chosen. The results of RQRM were clearly better than those of LRM. Consequently, eqs 5–8 were not used. The analysis showed that in this case, the LRM was not sufficient to model the experimental scenario, and more advanced modeling equations should be used, as provided herein by the RQRM.

**Table 8:** Comparison between Linear Regression Model and Reduced Quadratic Regression Model

	LRM			RQRM		
	R^2^	R^2^ (adj)	F-Value	R^2^	R^2^ (adj)	F-Value
σ_*B*_^*T*^ (*MPa*)	75.81%	74.18%	46.39	84.66%	82.43%	38.07
σ_*Y*_^*T*^ (*MPa*)	72.74%	70.90%	39.50	81.11%	78.37%	29.63
*E* ^*T*^ (*MPa*)	74.24%	72.50%	42.65	83.09%	80.63%	33.89
*T* ^*T*^ (*MJ*/*m* ^3^)	65.76%	63.44%	28.42	75.21%	71.62%	20.93

**LRM equations for σ**_***B***_^***T***^, **σ**_***Y***_^***T***^, ***E*** ^***T***^, ***T*** ^***T***^

5 $$\sigma_{B}^{T}=-59.9-0.1243\times RO+0.2955\times HT-1.144\times PV+0.2759\times IFR-0.1115\times DW$$

6 $$\sigma_{Y}^{T}=-69.2-0.1232\times RO+0.3099\times HT-1.053\times PV+0.2467\times IFR-0.1052\times DW$$

7 $$E^{T}=-545-0.2432\times RO+1.985\times HT-5.584\times PV+0.826\times IFR-0.3907\times DW$$

8 $$T^{T}=-1.40-0.01701\times RO+0.01902\times HT-0.0868\times PV+0.04091\times IFR-0.01394\times DW$$

### 3.4 Confirmation and Validation

Apart from the 16 experimental runs, two additional runs were conducted for validation purposes related to the prediction functions. The results are presented in Tables 9 and 10, which show the control parameters as well as the mean and standard deviation values for the measured responses, respectively. The Supporting Information also contains Table S2, which includes the results for all replicas of each confirmation run. The validation-derived data are presented in Table 11 in the form of experimental and predicted values along with the calculated absolute error, which remained low in all cases.

**Table 9:** Levels for the Confirmation Runs Control Parameters

**Run**	**RO (deg)**	**HT (°C)**	**PV** **(mm/s)**	**IFR (%)**	**DW (%)**
17	12	435	17	63	73
18	80	428	26	98	142

**Table 10:** Mean and Standard Deviations of the experimental Responses for σ_*B*_^*T*^, σ_*Y*_^*T*^, *E* ^*T*^, *T* ^*T*^, for the Confirmation Runs

**Run**	**σ**_***B***_^***T***^ **(** **MPa** **)**	**σ**_***B***_^***T***^ **(** **MPa** **)**	***E*** ^***T***^ **(** **MPa** **)**	***T*** ^***T***^ **(** ***MJ*** **/** **m** ^**3**^ **)**
17	62.08 ± 4.03	51.99 ± 4.25	263.09 ± 16.95	6.00 ± 0.41
18	43.34 ± 2.03	39.93 ± 2.80	184.21 ± 7.56	4.78 ± 0.30

**Table 11:** Validation Table

**Run**		**17**	**18**
Experimental	σ_*B*_^*T*^ (MPa)	62.08	43.34
	σ_*Y*_^*T*^ (MPa)	51.99	39.93
	*E* ^*T*^ (MPa)	263.09	184.21
	*T* ^*T*^ (MJ/m^3^)	6.00	4.78
Predicted	σ_*B*_^*T*^ (MPa)	58.02	39.18
	σ_*Y*_^*T*^ (MPa)	56.28	37.51
	*E* ^*T*^ (MPa)	239.22	169.49
	*T* ^*T*^ (MJ/m^3^)	6.30	4.41
Absolute Error	σ_*B*_^*T*^ (%)	6.54	9.60
	σ_*Y*_^*T*^ (%)	8.25	6.07
	*E* ^*T*^ (%)	9.07	7.99
	*T* ^*T*^ (%)	5.05	7.80

Figure 8 shows graphs where the *X*-axis contains the real data extracted from the experiments and the *Y*-axis contains the predicted data when applying the specifically selected settings for each experiment. Figure 8a is about σ_*B*_^*T*^, Figure 8b is about σ_*Y*_^*T*^, Figure 8c is about *E* ^*T*^ and Figure 8d is about *T* ^*T*^. Both the 16 experimental runs and two confirmations are included in the figures. The spots placed exactly on the diagonal line match the predicted and experimental results. For those placed in the right region, the predicted values were lower than the experimental values, whereas the opposite was true for those placed on the left side of the line. It appears that the majority of the values are very close to the line, which reveals a significant correlation between the predicted and the experimental results.

**Figure 8:**
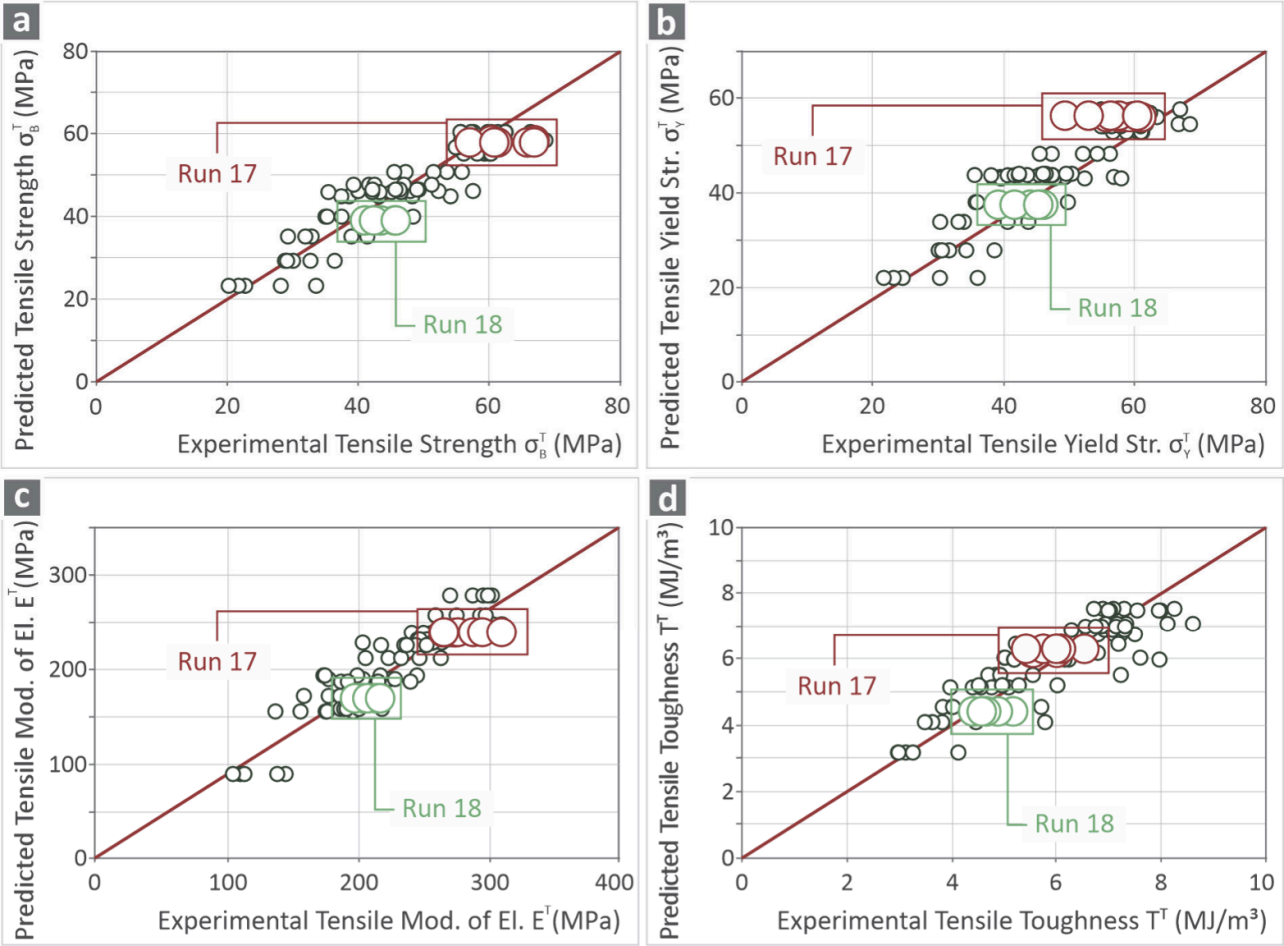
Correlation graphs about the predicted and experimental values of (a) σ_*B*_^*T*^, (b) σ_*Y*_^*T*^, (c) *E* ^*T*^ and (d) *T* ^*T*^ (runs 1–16 and confirmation runs 17–18).

Figure 9 presents the box plot graphs where the values for the two most important ranks are shown. The combinations used were σ_*B*_^*T*^ vs PV and IFR (Figure 9a), σ_*Y*_^*T*^ vs PV and IFR (Figure 9b), *E* ^*T*^ vs PV and HT (Figure 9c), and *T* ^*T*^ vs IFR and RO (Figure 9F). There are 16 (16) available runs of 16 (16) different combinations of all five (5) control parameters’ levels. Four (4) levels were kept from each of the controls, and all values belonging to the five (5) replicas are shown.

**Figure 9:**
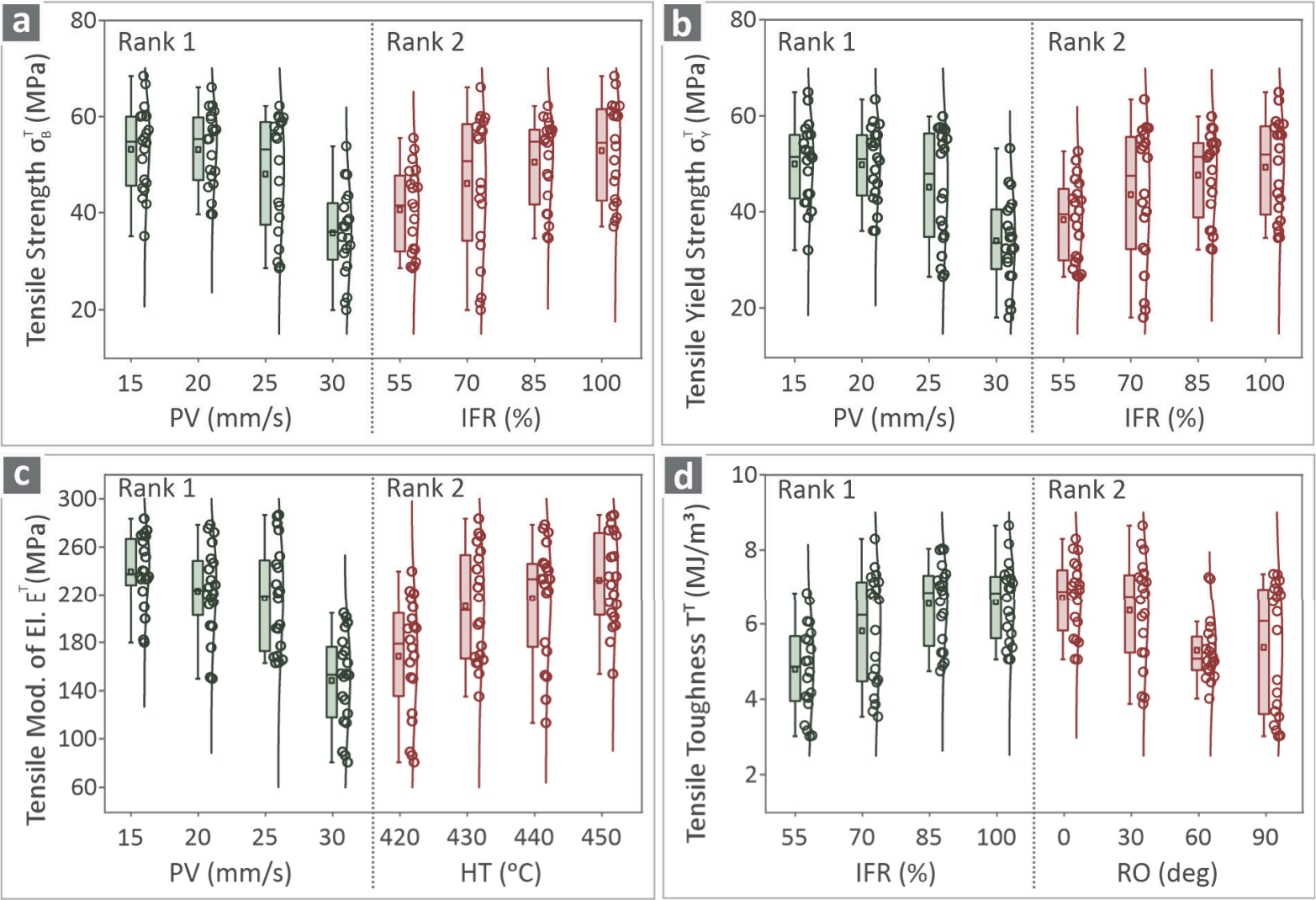
Box plots of (a) σ_*B*_^*T*^ vs PV and IFR, (b) σ_*Y*_^*T*^ vs PV and IFR, (c) *E* ^*T*^ vs PV and HT and (d) *T* ^*T*^ vs IFR and RO.

Figure 10 shows the surface graphs with two of the control parameters vs all the responses under investigation. Ranks 1 and 2 were chosen to be shown per response metric, whereas for σ_*B*_^*T*^ and σ_*Y*_^*T*^, Ranks 3 and 4 are also displayed. In particular, σ_*B*_^*T*^ versus PV and IFR (Figure 10a), σ_*Y*_^*T*^ versus PV and IFR (Figure 10b), *E* ^*T*^ versus PV and HT (Figure 10c), σ_*B*_^*T*^ versus RO and DW (Figure 10d), σ_*Y*_^*T*^ versus RO and DW (Figure 10e), and *T* ^*T*^ versus IFR and RO (Figure 10F). Each surface resulted from the RQRM equation, which was selected and solved in relation to the parameters belonging to ranks 1 and 2. For the remaining three parameters, the average value was kept constant for the calculation of each *z*-axis point on the surface.

**Figure 10:**
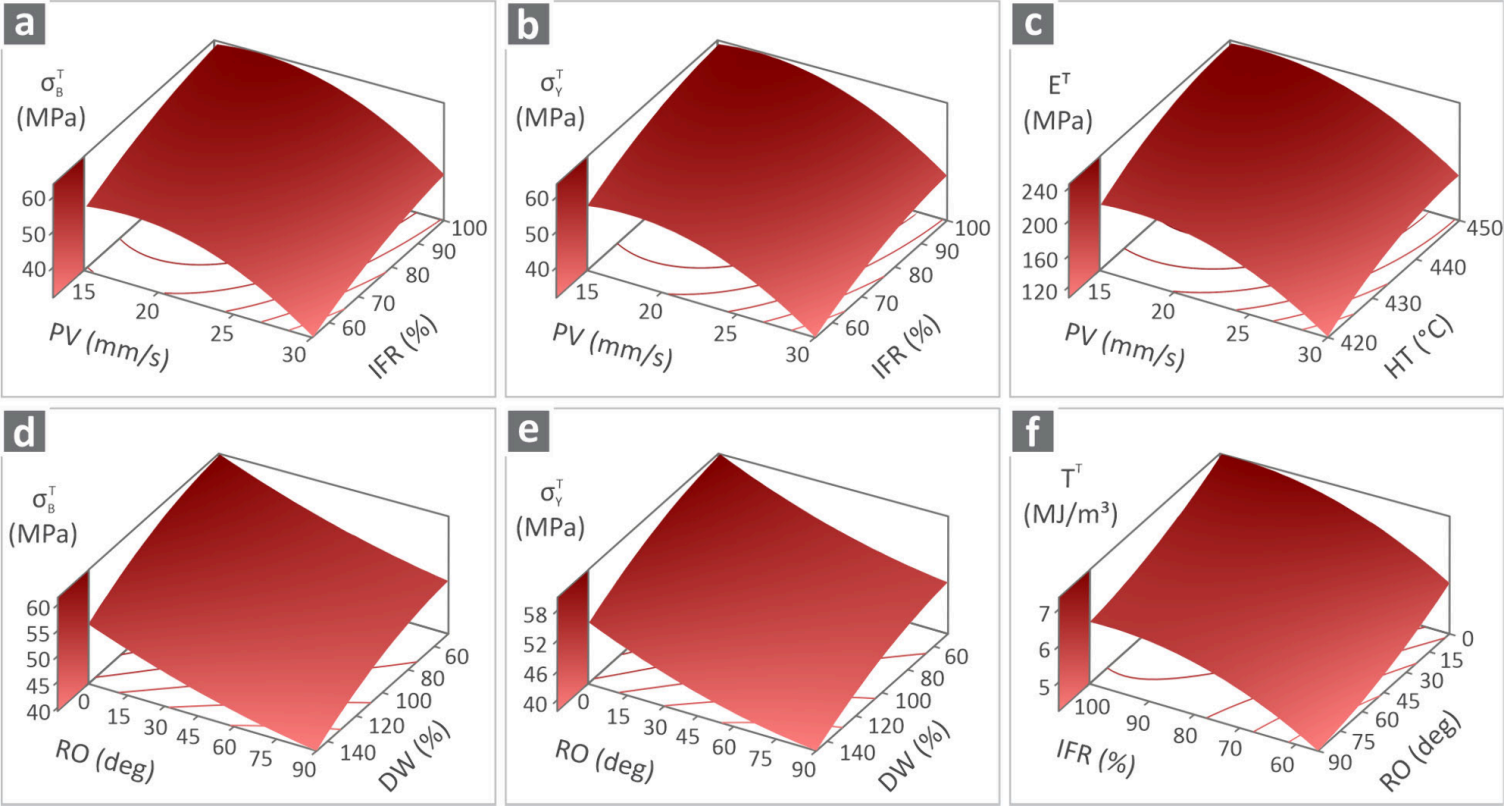
Surfaces of the examined responses versus the desired control parameters, namely (a) σ_*B*_^*T*^ vs PV and IFR, (b) σ_*Y*_^*T*^ vs PV and IFR, (c) *E* ^*T*^ vs PV and HT, (d) σ_*B*_^*T*^ vs RO and DW, (e) σ_*Y*_^*T*^ vs RO and DW, (f) *T* ^*T*^ vs IFR and RO.

## 4 Discussion

The Taguchi L16 design was used for the five (5) control parameters: RO, HT, PV, IFR, and DW. The chosen response metrics were related to the tensile properties, namely, σ_*B*_^*T*^, σ_*Y*_^*T*^, *E* ^*T*^ and *T* ^*T*^. The purpose of utilizing DOE is to reduce the number of required tests, manufacture PI specimens, and investigate their complexity. There were 16 (16) runs of five (5) replicas each, making a total (80) examined specimens, which would be much lower than the necessary specimens if a full factorial design were applied. This is a notable advancement regarding research of high-performance PI in MEX 3D printing. So far, in the literature, the mechanical properties of PI MEX 3D printed parts have been investigated for carbon fiber^120^ and carbon nanotubes^121^ composites. The first study considered print speed, layer thickness, and infill density without using any optimization modeling method, while the second study considered only nozzle temperature among the 3D printing parameters. Again, no statistical modeling tools were applied. Apart from these two studies, another research study focused on the filament preparation process, considering raster orientation and infill density from the 3D printing settings^122^ while in another work, only the infill density was considered.^123^ All four publications utilized a full factorial design approach with a limited number of specimens tested. Herein, five 3D printing parameters were simultaneously evaluated and ranked, while prediction models were introduced following a regression analysis. Their reliability was evaluated and is presented in the study.

The parameters were ranked based on the progress and behavior around the responses. In Rank 1, there were PV parameters for σ_*B*_^*T*^, σ_*Y*_^*T*^ and *E* ^*T*^, whereas for *T* ^*T*^, the IFR parameter was ranked 1. RO had the least important impact on *E* ^*T*^, whereas HT was ranked 5 for the rest of the responses. Based on the data provided in the respective tables, the highest levels of σ_*B*_^*T*^, σ_*Y*_^*T*^ and *T* ^*T*^ were detected in Run 6 (63.44 MPa, 59.87 MPa, and 7.76 MJ/m^3^, respectively). *E* ^*T*^ greatest levels appeared in Run 8 (275.55 MPa). Run 6 printing settings were RO: 30 deg, HT: 430 °C, PV: 15 mm/s, IFR: 100%, DW: 115%, and Run 8 printing settings were RO: 30 deg, HT: 450 °C, PV: 25 mm/s, IFR: 70%, DW: 55%. Surprisingly, the lowest levels of all responses were detected for Run 13 (σ_*B*_^*T*^: 24.97 MPa, σ_*Y*_^*T*^: 23.86 MPa, *E* ^*T*^: 98.48 MPa and *T* ^*T*^: 4.28 MJ/m^3^). Run 13 printing settings were namely RO: 90 deg, HT: 420 °C, PV: 30 mm/s, IFR: 70%, DW: 115%. For σ_*B*_^*T*^ there was a 254% difference in σ_*Y*_^*T*^ 251%), in *E* ^*T*^ 280%), and for *T* ^*T*^ 230%). These differences indicate the importance of the optimization method for discovering the best possible parameter combinations and managing the reinforcement properties.

Overall, the set of parameters that achieved better mechanical performance included a low raster orientation angle and print speed, median nozzle temperature, and 100% infill density. Regarding the raster orientation angle, aligning the printed filaments closer to the tensile loading axis facilitates improved load transfer along polymer chains and reduces stress concentration between the filaments. A slower print speed allows time for polymer chain diffusion (interlayer fusion) and minimizes the formation of voids between the filaments, thereby enhancing interfacial bonding. Printing at a median nozzle temperature mitigates the effects of under-extrusion at low temperatures. Furthermore, possible overheating at high temperatures is prevented, thus optimizing the polymer viscosity and molecular mobility for improved adhesion between filament layers. Finally, an infill density of 100% eliminates internal porosity, ensuring a continuous load-bearing structure.

From the MEP (Figure 7), printhead velocity was denoted as the most critical parameter (rank 1) in three of the four response metrics. Surprisingly, printhead velocity was ranked higher than the internal fill ratio in most metrics, as the internal fill ratio was ranked no. 2 in the strength metrics (ultimate and yield), no. 3 for stiffness, and no one, only for the toughness metric. Print speed is mainly related to the morphology of the parts and the fusion and adhesion of the strands. It can cause over- or under-extrusion issues with the filament during printing, and it has been reported to affect the polymer’s chain alignment,^124^ hence the effect of the fusion and adhesion of the strands and the morphology of the samples.^124,125^ Still, it has been reported that it does not highly affect the mechanical strength as much as other 3D printing parameters.^124,126^ On the other hand, infill density has been reported to highly affect the mechanical performance of parts. A clear trend is reported, with higher values being favorable to the mechanical performance in both tensile and flexural tests.^127,128^ As strength is affected by the materials area (and amount by extend), higher infill density increases the materials area for the same geometry and, as a result, the robustness of the part. Herein, for the specific high-performance polymer investigated, the analysis showed that the fusion between the layers, the polymer’s chain alignment, and the adhesion between the strands can have a greater effect on the mechanical performance of the parts than the higher amount of material in the part, which higher infill density provides.

However, all five control parameters clearly affected the response metrics. The increase in the raster orientation angle, printhead velocity, and deposition width reduced all four metrics, whereas the increase in the hot-end temperature and internal fill ratio increased all four metrics. These effects should be evaluated in combination with the rank of each control parameter in each response metric. Run 6, which achieved the best overall mechanical properties, had median HT and DW values, low PV and RO, and a high IFR. Run 13, which achieved the lowest overall mechanical properties, had median HT, DW, and IFR values and high PV and RO. Considering the highly ranked control parameters, it can be safely assumed that the median values for RO, HT, and DW, in combination with low PV and high IFR, can result in PI samples with higher mechanical properties.

Box plots (Figure 9) show that the whiskers differ (from small to large) between the levels of the control parameters, showing a higher variation in the values of the response metrics for specific values. Values are rather scattered in all cases, while there are also mild outliers, indicating that the data points are spread. Scattered data indicate a widespread, while data gathered around a specific value (which is not the case here) means the data set has a high concentration or frequency at that control parameter level. These findings should be evaluated in combination with MEP when selecting the control parameter levels for a 3D printing setting. Box plots show how each response metric varies for a specific control parameter value. On the other hand, MEP show how the variation of the values of each control parameter affect each response metric. Box plots and main effect plots assist in visualizing data, but they describe different functions. A box plot effectively summarizes a distribution (median, quartiles, range, etc.) - and has additional value by depicting outliers as well. Outliers are potentially interesting observations in order to find variability. A main effect plot is primarily associated with designed experiments or regression analysis. A primary distinction between box plots and main effect plots is that box plots look at the distribution and variability within groups or factors, whereas main effect plots look at the average impact a factor has on the response. By using box plots and main effect plots together, an overall understanding of the data and the influence of the factors on the response is provided.

Therefore, the trends presented in the MEP cannot be compared to those presented in the box plots. Both types of graphs provide useful information regarding the effect of the control parameters on the response metrics but in a different way. These two still are not enough for evaluating the control parameters’ impact on the response metrics. The control parameter ranking should also be considered, along with all the other parameters of the analysis presented. Furthermore, the experimental findings showed that a specific set of 3D printing setting values achieved the highest mechanical response. This shows that there are synergistic relations among the parameters. Box plots and MEP show part of the analysis and specific trends, but other parts of the analysis should be considered as well, as mentioned.

It is worth mentioning that the behavior of the tested samples changed from one parameter combination to another as both ductile and brittle specimens appeared during the capture of SEM images (Figure 5). Runs 2, 3, and 5, for example, appeared to be more brittle than Runs 1, 7, and 8, which were ductile. The differences in the brittleness of the samples between the runs are evident in Figure 4, in which the strain at which the samples failed differs between runs. This is expected to affect the toughness. For example, the sample from Run 6 failed at a higher strain than that from Run 11 (which seems to be the most brittle in the Figure). This, combined with higher strength and stiffness, resulted in the highest toughness values of the 16 runs. Run 13, which had the lowest strength and stiffness values, also had low toughness values, but was not the lowest among the runs, probably owing to a more ductile behavior, which is also evident in Figure 5. The lowest toughness was observed in the samples of Run 14, despite the high stiffness of the samples (a higher slope of the linear region of the curve). The rather low strength combined with brittle behavior (Figure 5), led to this outcome.

Furthermore, by examining the thermal properties of the high-performance PI (Figure 2), it was shown that the processing temperatures in the research did not cause any thermal degradation or were not close to the phase-changing temperatures, which might have affected the mechanical performance in the experiments.

The selection of the RQRM was beneficial, judging by the validated data (Table 11), comparing the experimental and predicted results, and considering the confirmation of Runs 17 and 18. In this specific experimental case, the LRM was proven to be less sufficient for modeling than RQRM, as the R^2^ values were approximately 70% for three of the response metrics and approximately 60% for the tensile toughness metric. Respectively, R^2^ values were approximately 10% for all four metrics. Again, the tensile toughness values were lower (∼70% instead of ∼ 80% for the other three metrics). Lower R^2^ values indicated that the model could predict the variability in the metric less accurately. F values were sufficiently high (>20) for all four metrics for both LRM and RQRM, showing strong evidence of the outcome. In the RQRM, the P values were statistically significant (*p* < 0.05) for all five control parameters for the tensile strength metric. For the other three response metrics, only the HT control metric had P-values of >0.05. However, only the tensile toughness was close (but lower) to one (0.822, consistent with the null hypothesis). The modeling process showed that the tensile toughness was more difficult to predict. However, when comparing the actual experimental data acquired in the confirmation run with the predicted values from the RQRM modeling process, the deviation was less than 10% for all four response metrics, verifying the accuracy of the prediction models.

The prediction models were not validated only through these two additional confirmation runs. Figure 8 shows graphs for all four response metrics, comparing the predicted values to the models and to the actual experimental values. All the experimental data derived are used to validate the predicted values of the response parameters. As shown, the values are close to the predicted values by the models. The values on the right side of the diagonal (red diagonal line) indicate that the model predicted lower values than the actual values. Therefore, the predicted values are safe. The values on the left side of the diagonal indicate that the model predicted higher values than the actual values, indicating that the actual strength was lower than the predicted value (on the safe side). The two additional confirmation runs, as shown, are on the right side of the diagonal, that is, on the safe side. Still, all values, as shown, are scattered around the prediction. Furthermore, the validity of the prediction models was evaluated through the calculated R^2^ values, which provided an indication of the accuracy and reliability of the prediction. The two additional confirmation runs were an excess safeguard step for the validation (not to say redundant). Two random sets of parameter values were selected (within the range studied in the research) to implement two additional runs and compare the results when the models had already been validated with all of the experimental data available in Figure 8. Regarding Figure 10, it visualizes the effect and trend that the two highly ranked control parameters for each response metric combined have. The slope and the shape of the 3D surfaces produced and presented in the graphs differ, visualizing the differences in the effect of the control parameters in each response metric.

## 5 Conclusions

The Taguchi L16 design was chosen for the optimization of the mechanical performance of high-performance PI 3D printed samples made by using the MEX method. RO, HT, PV, IFR, and DW are the control 3D printing parameters, and σ_*B*_^*T*^, σ_*Y*_^*T*^, *E* ^*T*^ and *T* ^*T*^ are the response metrics. It was revealed that investigating 3D printing settings at different levels to achieve enhanced specimen performance is of great importance and provides useful information. The optimization design, regression models, confirmation, and validation, as part of the research, have a significant impact and a vital role. Such findings justify the need for analysis, especially for high-performance polymers such as PI, operating in demanding environments and having high cost. These two critical aspects (the high cost of high-performance polymers, such as PI, and utilization in hazardous environments) highlight the merits of this research. The findings are summarized as follows:

- Mechanical tensile testing distinguished Run 6 (RO: 30 deg, HT: 430 °C, PV: 15 mm/s, IFR: 100%, DW: 115%) as the one having exceptional performance among the runs examined, as three out of the four responses were at their maximum levels in that case (the fourth one, Young’s modulus, was close to the highest value, too, with 268.91 MPa).
- Tensile strength improved 205% (Run 6, 63.44 MPa, vs 24.97 MPa), yield strength improved 251% (Run 6, 59.87 MPa vs 23.86 MPa), Young’s modulus improved 280% (Run 8, 275.55 MPa vs 98.48 MPa), and tensile toughness improved 230% (Run 6, 7.67Mj/m^3^ vs 3.3467Mj/m^3^).
- The microscopic evaluation of the samples provided important information about the effects of different parameter levels on specimen performance. In particular, changes were observed between the samples, some of which exhibited brittle behavior, such as Run 6, whereas others were ductile, such as Run 7.
- The importance of the PV parameter was highlighted (high values negatively affected the response metrics), as it was placed in Rank 1 for most of the responses, as was the IFR for one response metric.
- The HT and RO parameters did not significantly affect these responses.
- However, the LRM did not sufficiently model the experimental case. RQRM was proven more accurate, with R^2^ of approximately 80% for tensile strength, yield strength, and Young’s modulus, while for the tensile toughness R^2^ value was approximately 70%.
- The efficacy of the modeling process was proven, with less than 10% deviation between the predicted and the measured response metric values, constituting the models’ reliability for future use.

By considering the results of this research and using it to widen the related literature, PI utilization in various application fields can have growing potential and can prove to be significantly useful. The limitations of the research are related to the investigation of a single grade of PI and the mechanical testing only in uniaxial loading scenarios (tensile tests). Future studies can examine additional grades, implement tests in different loading scenarios, such as impact, flexural, or even dynamical loading tests, and finally expand the number of 3D printing parameters considered and the range of their levels to consider a wider area of applications.

## Supplementary Material



## Data Availability

The authors declare that the data supporting the findings of this study are available within the paper and its supplementary files.

## Notes

**Table S1**. Measured σ_*B*_^*T*^, σ_*Y*_^*T*^, *E* ^*T*^, *T* ^*T*^ for each experimental run, and five replicates per run; **Table S2**. Measured σ_*B*_^*T*^, σ_*Y*_^*T*^, *E* ^*T*^, *T* ^*T*^ for the five replicates of the confirmation experimental runs (PDF)

## References

[1] Vidakis N., Petousis M., Spyridaki M., Mountakis N., Dimitriou E., Michailidis N. (2026). Ultra- and High-Performance Polymers
for Material Extrusion Additive Manufacturing: Recent Advancements,
Challenges, and Optimization Perspectives. Mater.
Sci. Eng. R Rep..

[2] Tan L. J., Zhu W., Zhou K. (2020). Recent Progress on
Polymer Materials for Additive Manufacturing. Adv. Funct. Mater..

[3] El
Magri A., Vanaei S., Vaudreuil S. (2021). An Overview
on the Influence of Process Parameters through the Characteristic
of 3D-Printed PEEK and PEI Parts. High Perform.
Polym..

[4] Maloo L. M., Toshniwal S. H., Reche A., Paul P., Wanjari M. B. (2022). A Sneak
Peek Toward Polyaryletherketone (PAEK) Polymer: A Review. Cureus.

[5] Alqurashi H., Khurshid Z., Syed A. U. Y., Rashid Habib S., Rokaya D., Zafar M. S. (2021). Polyetherketoneketone (PEKK): An
Emerging Biomaterial for Oral Implants and Dental Prostheses. J. Adv. Res..

[6] Dallaev R., Pisarenko T., Sobola D., Orudzhev F., Ramazanov S., Trčka T. (2022). Brief Review of PVDF Properties and
Applications Potential. Polymers.

[7] Montagna, L. S. ; Kondo, M. Y. ; Callisaya, E. S. ; Mello, C. ; Souza, B. R. D. ; Lemes, A. P. ; Botelho, E. C. ; Costa, M. L. ; Alves, M. C. D. S. ; Ribeiro, M. V. ; Rezende, M. C. A Review on Research, Application, Processing, and Recycling of PPS Based Materials. Polímeros 2022, 32 (1). 10.1590/0104-1428.20210108.

[8] Wu W., Li Z., Lin G., Ma J., Gao Z., Qu H., Zhang F. (2022). Additive Manufacturing
of Continuous BF-Reinforced PES Composite
Material and Mechanical and Wear Properties Evaluation. J. Mater. Sci..

[9] Smith Z. J., Golias C. J., Vaske T. J., Young S. A., Chen Q., Goodbred L., Rong L., Cheng X., Penumadu D., Advincula R. C. (2024). Correlating
Viscosity and Die Swell in 3D Printing
of Polyphenylsulfone: A Thermo-Mechanical Optimization Modus Operandi. React. Funct. Polym..

[10] Tu R., Kim H. C., Sodano H. A. (2023). Additive
Manufacturing of High-Temperature
Thermoplastic Polysulfone with Tailored Microstructure via Precipitation
Printing. ACS Appl. Mater. Interfaces.

[11] Gouzman I., Grossman E., Verker R., Atar N., Bolker A., Eliaz N. (2019). Advances in Polyimide-Based Materials for Space Applications. Adv. Mater..

[12] Weyhrich C. W., Long T. E. (2022). Additive Manufacturing of High-performance Engineering
Polymers: Present and Future. Polym. Int..

[13] Chen P., Wang H., Su J., Tian Y., Wen S., Su B., Yang C., Chen B., Zhou K., Yan C., Shi Y. (2022). Recent Advances
on High-Performance Polyaryletherketone Materials
for Additive Manufacturing. Adv. Mater..

[14] Dua R., Rashad Z., Spears J., Dunn G., Maxwell M. (2021). Applications
of 3D-Printed PEEK via Fused Filament Fabrication: A Systematic Review. Polymers.

[15] Qian B., Ji K., Lu W., Wu G., Tan B., Jing J., Ji J. (2025). Polyetherketoneketone, a High-Performance Polymer for Splinting Mobile
Teeth: A Clinical Report. J. Prosthet. Dent..

[16] Zol S. M., Alauddin M. S., Said Z., Mohd Ghazali M. I., Hao-Ern L., Mohd Farid D. A., Zahari N. A. H., Al-Khadim A. H. A., Abdul Aziz A. H. (2023). Description
of Poly­(Aryl-Ether-Ketone)
Materials (PAEKs), Polyetheretherketone (PEEK) and Polyetherketoneketone
(PEKK) for Application as a Dental Material: A Materials Science Review. Polymers.

[17] Molinar-Díaz J., Parsons A. J., Ahmed I., Warrior N. A., Harper L. T. (2025). Poly-Ether-Ether-Ketone
(PEEK) Biomaterials and Composites: Challenges, Progress, and Opportunities. Polym. Rev..

[18] Chen Z., Chen Y., Wang Y., Deng J., Wang X., Wang Q., Liu Y., Ding J., Yu L. (2023). Polyetheretherketone
Implants with Hierarchical Porous Structure for Boosted Osseointegration. Biomater. Res..

[19] Rinaldi M., Ferrara M., Pigliaru L., Allegranza C., Nanni F. (2023). Additive Manufacturing of Polyether Ether Ketone-Based Composites
for Space Application: A Mini-Review. CEAS Space
J..

[20] Rinaldi M., Cecchini F., Pigliaru L., Ghidini T., Lumaca F., Nanni F. (2021). Additive Manufacturing of Polyether
Ether Ketone (PEEK) for Space
Applications: A Nanosat Polymeric Structure. Polymers.

[21] Ling X., Jing X., Zhang C., Chen S. (2020). Polyether Ether Ketone
(PEEK) Properties and Its Application Status. IOP Conf. Ser. Earth Environ. Sci..

[22] Thiruchitrambalam M., Bubesh Kumar D., Shanmugam D., Jawaid M. (2020). A Review on PEEK Composites
– Manufacturing Methods, Properties and Applications. Mater. Today Proc..

[23] Lu C., Xu N., Zheng T., Zhang X., Lv H., Lu X., Xiao L., Zhang D. (2019). The Optimization of Process Parameters
and Characterization of High-Performance CF/PEEK Composites Prepared
by Flexible CF/PEEK Plain Weave Fabrics. Polymers.

[24] Gupta R., Shinde S., Yella A., Subramaniam C., Saha S. K. (2020). Thermomechanical Characterisations
of PTFE, PEEK, PEKK
as Encapsulation Materials for Medium Temperature Solar Applications. Energy.

[25] Badeghaish W., Wagih A., Rastogi S., Lubineau G. (2024). Effect of
High-Temperature
Acid Aging on Microstructure and Mechanical Properties of PEEK. Polym. Test..

[26] Market Research Future. Global High Performance Polymer Market Overview Source *:* https://www.marketresearchfuture.com/reports/high-performance-polymer-market-36287.

[27] insightaceanalytic. High-Performance Polymers Market Size, Share & Trends Analysis Report By Type (Fluoropolymers, Polyphthalamide (PPA), Polyphenylene Sulphide (PPS), Sulfone Polymers (SP), Polyamide (PA), Polyketones, Polyimides, Liquid Crystal Polymers (LCP)), By End-User Industry (Automobile, Aerospace & Defense, Electrical & Electronics, Industrial, Medical), Region And Segment Forecasts, 2024–2031. https://www.insightaceanalytic.com/report/high-performance-polymers-market/2117.

[28] Emergen Research . High-Performance Polymers (HPPS) Market, By Product (Polyphthalamide (PPA), Polyphenylene Sulfide (PPS), and Others), By Application (Automotive, Medical, and Others), and By Region Forecast to 2032. https://www.emergenresearch.com/industry-report/high-performance-polymers-market?srsltid=AfmBOor3kENtoS-bnLruttrvtxQ-D1HpEbqwH-TkGfJ_-K_UudtLL4aF.

[29] Grand View Research . High Performance Plastics Market Size, Share & Trends Analysis Report By Product (Fluoropolymers, Polyamides), By Application (Transportation, Electrical & Electronics), By Region, And Segment Forecasts, 2025 - 2030. https://www.grandviewresearch.com/industry-analysis/high-performance-plastics-market-report.

[30] Lin J., Su J., Weng M., Xu W., Huang J., Fan T., Liu Y., Min Y. (2022). Applications
of Flexible Polyimide: Barrier Material,
Sensor Material, and Functional Material. Soft
Sci..

[31] Zhang Z., Wang X., Zu G., Kanamori K., Nakanishi K., Shen J. (2019). Resilient, Fire-Retardant
and Mechanically Strong Polyimide-Polyvinylpolymethylsiloxane
Composite Aerogel Prepared via Stepwise Chemical Liquid Deposition. Mater. Des..

[32] Ke H., Zhao L., Zhang X., Qiao Y., Wang G., Wang X. (2020). Performance of High-Temperature Thermosetting Polyimide Composites
Modified with Thermoplastic Polyimide. Polym.
Test..

[33] Duan C., He R., Li S., Yang R., Shao M., Yang Z., Zhang N., Yuan P., Wang T., Wang Q. (2020). Effect of
Atomic Oxygen on Corrosion and Friction and Wear Behavior of Polyimide
Composites. J. Appl. Polym. Sci..

[34] Guo Y., Lyu Z., Yang X., Lu Y., Ruan K., Wu Y., Kong J., Gu J. (2019). Enhanced Thermal
Conductivities and
Decreased Thermal Resistances of Functionalized Boron Nitride/Polyimide
Composites. Compos. Part B Eng..

[35] Li Y., Sun G., Zhou Y., Liu G., Wang J., Han S. (2022). Progress in
Low Dielectric Polyimide Film – A Review. Prog. Org. Coat..

[36] Ma S., Wang S., Jin S., Wang Y., Yao J., Zhao X., Chen C. (2020). Construction
of High-Performance,
High-Temperature Shape Memory Polyimides Bearing Pyridine and Trifluoromethyl
Group. Polymer.

[37] Liu Y., Wang Y., Wu D. (2022). Synthetic
Strategies for Highly Transparent
and Colorless Polyimide Film. J. Appl. Polym.
Sci..

[38] Sezer
Hicyilmaz A., Celik Bedeloglu A. (2021). Applications of Polyimide Coatings:
A Review. SN Appl. Sci..

[39] Wu X., Liu J., Jiang G., Zhang Y., Guo C., Zhang Y., Qi L., Zhang X. (2019). Highly Transparent Preimidized Semi-Alicyclic Polyimide
Varnishes with Low Curing Temperatures and Desirable Processing Viscosities
at High Solid Contents: Preparation and Applications for LED Chip
Passivation. J. Mater. Sci. Mater. Electron..

[40] Hong M., Lee S., Choi S., Mun J. (2020). Polyimide Binder for a High-Energy-Density
Composite Anode Electrode with Graphite and Silicon. J. Electroanal. Chem..

[41] Jeong H., Lee T., Kim J., Jeong H. S., Jun S. B., Seo J.-M. (2024). Fabrication
and Validation of Flexible Neural Electrodes Based on Polyimide Tape
and Gold Sheet. Biomed. Eng. Lett..

[42] Zhu C., Xue T., Ma Z., Fan W., Liu T. (2023). Mechanically Strong
and Thermally Insulating Polyimide Aerogel Fibers Reinforced by Prefabricated
Long Polyimide Fibers. ACS Appl. Mater. Interfaces.

[43] Li X., Zhang B., Wu Z., Liu Y., Hu J., Zhang C., Cao G., Zhang K., Sun J., Liu X., Xu W. (2023). Highly Flexible,
Large Scaled and Electrical Insulating
Polyimide Composite Paper with Nanoscale Polyimide Fibers. Compos. Commun..

[44] Liu H., Wang X., Antwi-Afari M. F., Mi H.-Y., Liu C. (2024). A State-of-the-Art
Review of Polyimide Foams Research. Constr.
Build. Mater..

[45] Liu X.-J., Zheng M.-S., Chen G., Dang Z.-M., Zha J.-W. (2022). High-Temperature
Polyimide Dielectric Materials for Energy Storage: Theory, Design,
Preparation and Properties. Energy Environ.
Sci..

[46] Zhu Y., Zhu Y., Huang X., Chen J., Li Q., He J., Jiang P. (2019). High Energy Density Polymer Dielectrics Interlayered by Assembled
Boron Nitride Nanosheets. Adv. Energy Mater..

[47] Pei J.-Y., Yin L.-J., Zhong S.-L., Dang Z.-M. (2023). Suppressing the
Loss of Polymer-Based Dielectrics for High Power Energy Storage. Adv. Mater..

[48] Wu Z., He J., Yang H., Yang S. (2022). Progress in Aromatic Polyimide Films
for Electronic Applications: Preparation, Structure and Properties. Polymers.

[49] Ding L., Yu Z.-D., Wang X.-Y., Yao Z.-F., Lu Y., Yang C.-Y., Wang J.-Y., Pei J. (2023). Polymer Semiconductors:
Synthesis, Processing, and Applications. Chem.
Rev..

[50] Naqi M., Kim B., Kim S., Kim S. (2021). Pulsed Gate
Switching of MoS_2_ Field-Effect Transistor Based on Flexible
Polyimide Substrate
for Ultrasonic Detectors. Adv. Funct. Mater..

[51] Zhang J.-H., Li Z., Xu J., Li J., Yan K., Cheng W., Xin M., Zhu T., Du J., Chen S., An X., Zhou Z., Cheng L., Ying S., Zhang J., Gao X., Zhang Q., Jia X., Shi Y., Pan L. (2022). Versatile
Self-Assembled Electrospun Micropyramid Arrays for High-Performance
on-Skin Devices with Minimal Sensory Interference. Nat. Commun..

[52] Wan B., Dong X., Yang X., Wang J., Zheng M., Dang Z., Chen G., Zha J. (2023). Rising of Dynamic Polyimide
Materials: A Versatile Dielectric for Electrical and Electronic Applications. Adv. Mater..

[53] Shu J., Zhou Z., Liang H., Yang X. (2024). Polyimide as a Biomedical
Material: Advantages and Applications. Nanoscale
Adv..

[54] Baumann S., Stone R. T., Abdelall E. (2025). Smart Healthcare at Home: A Review
of AI-Enabled Wearables and Diagnostics Through the Lens of the Pi-CON
Methodology. Sensors.

[55] Lee D. U., Kim D. W., Lee S. Y., Choi D. Y., Choi S. Y., Moon K.-S., Shon M. Y., Moon M. J. (2022). Amino Acid-Mediated
Negatively Charged Surface Improve Antifouling and Tribological Characteristics
for Medical Applications. Colloids Surf. B Biointerfaces.

[56] Vargason A. M., Anselmo A. C., Mitragotri S. (2021). The Evolution
of Commercial Drug
Delivery Technologies. Nat. Biomed. Eng..

[57] Kim D.-S., Jeong Y.-J., Shanmugasundaram A., Oyunbaatar N.-E., Park J., Kim E.-S., Lee B.-K., Lee D.-W. (2021). 64 PI/PDMS
Hybrid Cantilever Arrays with an Integrated Strain Sensor for a High-Throughput
Drug Toxicity Screening Application. Biosens.
Bioelectron..

[58] Pagneux Q., Ye R., Chengnan L., Barras A., Hennuyer N., Staels B., Caina D., Osses J. I. A., Abderrahmani A., Plaisance V., Pawlowski V., Boukherroub R., Melinte S., Szunerits S. (2020). Electrothermal Patches Driving the
Transdermal Delivery of Insulin. Nanoscale Horiz..

[59] Unver N., Odabas S., Demirel G. B., Gul O. T. (2022). Hollow Microneedle
Array Fabrication Using a Rational Design to Prevent Skin Clogging
in Transdermal Drug Delivery. J. Mater. Chem.
B.

[60] Zhao C., Man T., Cao Y., Weiss P. S., Monbouquette H. G., Andrews A. M. (2022). Flexible and Implantable Polyimide Aptamer-Field-Effect
Transistor Biosensors. ACS Sens..

[61] Panda B., Mandal S., Majerus S. J. A. (2019). Flexible, Skin Coupled Microphone
Array for Point of Care Vascular Access Monitoring. IEEE Trans. Biomed. Circuits Syst..

[62] Herbert R., Mishra S., Lim H., Yoo H., Yeo W. (2019). Fully Printed,
Wireless, Stretchable Implantable Biosystem toward Batteryless, Real-Time
Monitoring of Cerebral Aneurysm Hemodynamics. Adv. Sci..

[63] Ling Y., Pang W., Liu J., Page M., Xu Y., Zhao G., Stalla D., Xie J., Zhang Y., Yan Z. (2022). Bioinspired Elastomer Composites with Programmed Mechanical and Electrical
Anisotropies. Nat. Commun..

[64] Kaewmanee R., Wang F., Mei S., Pan Y., Yu B., Wu Z., Meesane J., Wei J. (2022). Molybdenum
Disulfide Nanosheet/Polyimide
Composites with Improved Tribological Performances, Surface Properties,
Antibacterial Effects and Osteogenesis for Facilitating Osseointegration. J. Mater. Chem. B.

[65] Zhang Y., Jiang W., Yuan S., Zhao Q., Liu Z., Yu W. (2020). Impacts of a Nano-Laponite
Ceramic on Surface Performance, Apatite
Mineralization, Cell Response, and Osseointegration of a Polyimide-Based
Biocomposite. Int. J. Nanomedicine.

[66] Kaewmanee R., Wang F., Pan Y., Mei S., Meesane J., Li F., Wu Z., Wei J. (2022). Microporous
Surface Containing Flower-like
Molybdenum Disulfide Submicro-Spheres of Sulfonated Polyimide with
Antibacterial Effect and Promoting Bone Regeneration and Osteointegration. Biomater. Sci..

[67] Zhong H., Zhu Z., You P., Lin J., Cheung C. F., Lu V. L., Yan F., Chan C.-Y., Li G. (2020). Plasmonic and Superhydrophobic Self-Decontaminating
N95 Respirators. ACS Nano.

[68] Mariello M., Qualtieri A., Mele G., De Vittorio M. (2021). Metal-Free
Multilayer Hybrid PENG Based on Soft Electrospun/-Sprayed Membranes
with Cardanol Additive for Harvesting Energy from Surgical Face Masks. ACS Appl. Mater. Interfaces.

[69] Zhang Y., Huang Z., Ruan B., Zhang X., Jiang T., Ma N., Tsai F. (2020). Design and
Synthesis of Polyimide Covalent Organic
Frameworks. Macromol. Rapid Commun..

[70] Bi H., Zhi X., Wu P., Zhang Y., Wu L., Tan Y., Jia Y.-J., Liu J., Zhang X. (2020). Preparation and Characterization
of Semi-Alicyclic Polyimide Resins and the Derived Alignment Layers
for Liquid Crystal Display Technology. Polymers.

[71] Choi K., Droudian A., Wyss R. M., Schlichting K.-P., Park H. G. (2018). Multifunctional Wafer-Scale Graphene
Membranes for
Fast Ultrafiltration and High Permeation Gas Separation. Sci. Adv..

[72] Research and Markets . Polyimide Market Size, Share & Trends Analysis Report by Type (Polyimide Resin, Polyimide Film, Polyimide Coatings, Polyimide Varnish), by Application (Medical, Energy & Power), by Region, and Segment Forecasts, 2025–2030. https://www.researchandmarkets.com/report/polyimide?srsltid=AfmBOoratQOCCdUQAznR_C3sHq9M7ZLHr8fWy44GAmWF0cRRAcaTUuyz.

[73] Weyhrich C. W., Long T. E. (2022). Additive Manufacturing
of High-performance Engineering
Polymers: Present and Future. Polym. Int..

[74] Haidiezul A. H. M., Hazwan M. H. M., Soon Lee W., Gunalan, Fatin Najihah N., Fadhli I. (2020). Full Factorial Design
Exploration Approach for Multi-Objective
Optimization on the (FDM) 3D Printed Part. IOP
Conf. Ser. Mater. Sci. Eng..

[75] Vidakis N., Petousis M., Mountakis N., Kechagias J. D. (2022). Material
Extrusion 3D Printing and Friction Stir Welding : An Insight
into the Weldability of Polylactic Acid Plates Based on a Full Factorial
Design. Int. J. Adv. Manuf. Technol..

[76] Ballantyne K. N., Van Oorschot R. A., Mitchell R. J. (2008). Reduce Optimisation Time and Effort:
Taguchi Experimental Design Methods. Forensic
Sci. Int. Genet. Suppl. Ser..

[77] David C., Sagris D., Petousis M., Nasikas N. K., Moutsopoulou A., Sfakiotakis E., Mountakis N., Charou C., Vidakis N. (2023). Operational
Performance and Energy Efficiency of MEX 3D Printing with Polyamide
6 (PA6): Multi-Objective Optimization of Seven Control Settings Supported
by L27 Robust Design. Appl. Sci..

[78] Petousis M., Vidakis N., Mountakis N., Karapidakis E., Moutsopoulou A. (2023). Functionality Versus Sustainability
for PLA in MEX
3D Printing : The Impact of Generic Process Control Factors
on Flexural Response and Energy Efficiency. Polymers.

[79] Petousis M., Spiridaki M., Mountakis N., Moutsopoulou A., Maravelakis E., Vidakis N. (2024). Box-Behnken Modeling to Optimize
the Engineering Response and the Energy Expenditure in Material Extrusion
Additive Manufacturing of Short Carbon Fiber Reinforced Polyamide
6. Int. J. Adv. Manuf. Technol..

[80] Vidakis N., Petousis M., Mountakis N., Karapidakis E. (2023). Box-Behnken
Modeling to Quantify the Impact of Control Parameters on the Energy
and Tensile Efficiency of PEEK in MEX 3D-Printing. Heliyon.

[81] Vidakis N., Petousis M., Mountakis N., Papadakis V., Moutsopoulou A. (2023). Mechanical Strength Predictability of Full Factorial,
Taguchi, and Box Behnken Designs: Optimization of Thermal Settings
and Cellulose Nanofibers Content in PA12 for MEX AM. J. Mech. Behav. Biomed. Mater..

[82] Chueca
De Bruijn A., Gómez-Gras G., Fernández-Ruano L., Farràs-Tasias L., Pérez M. A. (2023). Optimization of a Combined Thermal
Annealing and Isostatic Pressing Process for Mechanical and Surface
Enhancement of Ultem FDM Parts Using Doehlert Experimental Designs. J. Manuf. Process..

[83] Jabeur M., Souissi S., Jerbi A., Elloumi A. (2025). Optimization
of FDM
3D Printing Parameters of PLA and Composite Materials Using Definitive
Screening Design. Int. J. Adv. Manuf. Technol..

[84] Gregor M., Grznar P., Mozol S., Mozolova L. (2024). PLACKETT-BURMAN DESIGN. Acta
Simulatio.

[85] Vidakis N., Petousis M., Mountakis N., Moutsopoulou A., Karapidakis E. (2023). Energy Consumption vs. Tensile Strength of Poly [ Methyl
Methacrylate ] in Material Extrusion 3D Printing : The Impact
of Six Control Settings. Polymers.

[86] Vidakis N., Petousis M., David C. N., Sagris D., Mountakis N., Karapidakis E. (2023). Mechanical
Performance over Energy Expenditure in MEX
3D Printing of Polycarbonate: A Multiparametric Optimization with
the Aid of Robust Experimental Design. J. Manuf.
Mater. Process..

[87] Petousis M., Mountakis N., Spyridaki M., Gkagkanatsiou K., Valsamos I., Moutsopoulou A., Stratakis E., Vidakis N. (2025). Process Optimization of Material Extrusion Additive
Manufacturing with ASA: Robust Design and Predictive Models for Engineering
Response Metrics. Int. J. Adv. Manuf. Technol..

[88] Vidakis N., Petousis M., Nasikas N. K., Manios T., Mountakis N., Valsamos J., Sfakiotakis E. (2024). Optimization
of the Engineering Response
of Medical-Graded Polycaprolactone (PCL) over Multiple Generic Control
Parameters in Bioplotting. Int. J. Adv. Manuf.
Technol..

[89] Hikmat M., Rostam S., Ahmed Y. M. (2021). Investigation
of Tensile Property-Based
Taguchi Method of PLA Parts Fabricated by FDM 3D Printing Technology. Results Eng..

[90] Tunçel O., Tüfekci K., Kahya Ç. (2024). Multi-Objective
Optimization of 3D
Printing Process Parameters Using Gray-Based Taguchi for Composite
PLA Parts. Polym. Compos..

[91] Chyr G., DeSimone J. M. (2023). Review of High-Performance
Sustainable Polymers in
Additive Manufacturing. Green Chem..

[92] Jiang C.-P., Cheng Y.-C., Lin H.-W., Chang Y.-L., Pasang T., Lee S.-Y. (2022). Optimization of
FDM 3D Printing Parameters for High
Strength PEEK Using the Taguchi Method and Experimental Validation. Rapid Prototyp. J..

[93] Vidakis N., Petousis M., David C., Sagris D., Mountakis N., Spiridaki M., Moutsopoulou A., Nasikas N. K. (2024). Robust Design Optimization
of Critical Quality Indicators (CQIs) of Medical-Graded Polycaprolactone
(PCL) in Bioplotting. Bioprinting.

[94] Petousis M., Mountakis N., Vidakis N. (2023). Optimization of Hybrid Friction Stir
Welding of PMMA: 3D-Printed Parts and Conventional Sheets Welding
Efficiency in Single- and Two-Axis Welding Traces. Int. J. Adv. Manuf. Technol..

[95] Vidakis N., David C. N., Petousis M., Sagris D., Mountakis N. (2023). Optimization
of Key Quality Indicators in Material Extrusion 3D Printing of Acrylonitrile
Butadiene Styrene: The Impact of Critical Process Control Parameters
on the Surface Roughness, Dimensional Accuracy, and Porosity. Mater. Today Commun..

[96] Mountakis N., Nasikas N. K., Moutsopoulou A., David C., Petousis M., Vidakis N. (2025). Friction Stir Welding of ASA Additive Manufactured
with MEX Extrusion: Weldability and Process Optimization. Weld. Int..

[97] Rashed K., Kafi A., Simons R., Bateman S. (2023). Optimization of Material
Extrusion Additive Manufacturing Process Parameters for Polyether
Ketone Ketone (PEKK). Int. J. Adv. Manuf. Technol..

[98] Retolaza J., Gondra K., Ansola R., Allue A. (2022). Mechanical
Research
to Optimize Parameter Selection for PPS Material Processed by FDM. Mater. Manuf. Process..

[99] Mohamed T., Barhoumi N., Lamnawar K., Maazouz A., Znaidi A. (2021). Optimization
of Fused Deposition Modeling Process Parameters Using the Taguchi
Method to Improve the Tensile Properties of 3D-Printed Polyether Ether
Ketone. Proc. Inst. Mech. Eng. Part J. Mater.
Des. Appl..

[100] Borah J., Chandrasekaran M., Selvarajan L. (2025). Taguchi-Based
Experimental Investigation and Modeling of 3D-Printed PEEK Parts as
Biomedical Implants Using Fused Deposition Modeling for Improving
Mechanical Strength and Surface Quality. J.
Mater. Eng. Perform..

[101] Vidakis N., Petousis M., Spiridaki M., Mountakis N., Moutsopoulou A., Kymakis E. (2024). Optimization of Critical
Process Control Parameters in MEX Additive Manufacturing of High-Performance
Polyethylenimine: Energy Expenditure, Mechanical Expectations, and
Productivity Aspects. Int. J. Adv. Manuf. Technol..

[102] Vidakis N., Petousis M., David C., Nasikas N. K., Sagris D., Mountakis N., Spiridaki M., Moutsopoulou A., Stratakis E. (2025). Critical Quality
Indicators of High-Performance
Polyetherimide (ULTEM) over the MEX 3D Printing Key Generic Control
Parameters: Prospects for Personalized Equipment in the Defense Industry. Def. Technol..

[103] Liu H., Cheng X., Yang X. H., Zheng G. M., Guo Q. J. (2019). Experimental
Study on Parameters of 3D Printing Process for PEEK Materials. IOP Conf. Ser. Mater. Sci. Eng..

[104] Zhang J., Zhang Y., Tao L., Wang T., Wang Q. (2023). Integrated Printing of High-Strength,
High-Shape-Retaining Polyimide
and Its Composite Gradient Structures for Enhanced Tribological Properties. Addit. Manuf..

[105] Niu B., Shi M., Zhang Z., Li Y., Cao Y., Pan S. (2022). Multi-Objective Optimization of Supply Air Jet Enhancing
Airflow
Uniformity in Data Center with Taguchi-Based Grey Relational Analysis. Build. Environ..

[106] Arslanoglu N., Yigit A. (2016). Experimental Investigation
of Radiation
Effect on Human Thermal Comfort by Taguchi Method. Appl. Therm. Eng..

[107] Chang C.-W., Kuo C.-P. (2007). Evaluation of Surface
Roughness in
Laser-Assisted Machining of Aluminum Oxide Ceramics with Taguchi Method. Int. J. Mach. Tools Manuf..

[108] Pinar A. M., Uluer O., Kırmaci V. (2009). Optimization
of Counter Flow Ranque–Hilsch Vortex Tube Performance Using
Taguchi Method. Int. J. Refrig..

[109] Özel S., Vural E., Binici M. (2020). Optimization of the
Effect of Thermal Barrier Coating (TBC) on Diesel Engine Performance
by Taguchi Method. Fuel.

[110] Tutar M., Aydin H., Yuce C., Yavuz N., Bayram A. (2014). The Optimisation of Process Parameters
for Friction
Stir Spot-Welded AA3003-H12 Aluminium Alloy Using a Taguchi Orthogonal
Array. Mater. Des..

[111] Yang W. H., Tarng Y. S. (1998). Design Optimization
of Cutting Parameters
for Turning Operations Based on the Taguchi Method. J. Mater. Process. Technol..

[112] Ning M., Mengjie S., Mingyin C., Dongmei P., Shiming D. (2016). Computational Fluid Dynamics (CFD)
Modelling of Air
Flow Field, Mean Age of Air and CO2 Distributions inside a Bedroom
with Different Heights of Conditioned Air Supply Outlet. Appl. Energy.

[113] Arslanoglu N., Yigit A. (2017). Investigation of Efficient
Parameters
on Optimum Insulation Thickness Based on theoretical-Taguchi Combined
Method. Environ. Prog. Sustain. Energy.

[114] Simpson J.
R. (1996). Taguchi Techniques for
Quality Engineering. J. Qual. Technol..

[115] Bademlioglu A. H., Canbolat A. S., Yamankaradeniz N., Kaynakli O. (2018). Investigation of Parameters
Affecting Organic Rankine
Cycle Efficiency by Using Taguchi and ANOVA Methods. Appl. Therm. Eng..

[116] Soni A., Patel R. M., Kumar K., Pareek K. (2022). Optimization
for Maximum Extraction of Solder from Waste PCBs through Grey Relational
Analysis and Taguchi Technique. Miner. Eng..

[117] Palanikumar K. (2011). Experimental Investigation and Optimisation
in Drilling
of GFRP Composites. Measurement.

[118] Ri R. H., Yang W.-C. (2025). Optimization of the Metal Injection
Molding Process with 3l6L Stainless Steel Powder and Influence Analysis
of Process Parameters Using the Taguchi-MADM-Based Hybrid Method. ACS Omega.

[119] Mishra S. S., Mohapatra T., Sahoo S. S., Mishra P. (2022). Overall Performance
Investigation and Optimization of a Multi-Fuel Operated Compression
Ignition Engine Using Coupled Taguchi and Grey Relational Analysis. ACS Omega.

[120] Ye W., Lin G., Wu W., Geng P., Hu X., Gao Z., Zhao J. (2019). Separated
3D Printing of Continuous Carbon Fiber Reinforced
Thermoplastic Polyimide. Compos. Part Appl.
Sci. Manuf..

[121] Ye W., Wu W., Hu X., Lin G., Guo J., Qu H., Zhao J. (2019). 3D Printing of Carbon Nanotubes Reinforced Thermoplastic
Polyimide Composites with Controllable Mechanical and Electrical Performance. Compos. Sci. Technol..

[122] Abbott A., Gibson T., Tandon G. P., Hu L., Avakian R., Baur J., Koerner H. (2021). Melt Extrusion and
Additive Manufacturing of a Thermosetting Polyimide. Addit. Manuf..

[123] Kothavade P., Kafi A., Dekiwadia C., Kumar V., Sukumaran S. B., Shanmuganathan K., Bateman S. (2024). Extrusion 3D Printing of Intrinsically Fluorescent
Thermoplastic Polyimide: Revealing an Undisclosed Potential. Polymers.

[124] Loskot J., Jezbera D., Loskot R., Bušovský D., Barylski A., Glowka K., Duda P., Aniołek K., Voglová K., Zubko M. (2023). Influence of Print Speed on the Microstructure,
Morphology, and Mechanical Properties of 3D-Printed PETG Products. Polym. Test..

[125] da Silva D. P., Pinheiro J., Abdulghani S., Kamma Lorger C., Martinez J. C., Solano E., Mateus A., Pascoal-Faria P., Mitchell G. R. (2022). Changing the Paradigm-Controlling
Polymer Morphology during 3D Printing Defines Properties. Polymers.

[126] Afonso J. A., Alves J. L., Caldas G., Gouveia B. P., Santana L., Belinha J. (2021). Influence of 3D Printing Process
Parameters on the Mechanical Properties and Mass of PLA Parts and
Predictive Models. Rapid Prototyp. J..

[127] Karad A. S., Sonawwanay P. D., Naik M., Thakur D. G. (2023). Experimental
Study of Effect of Infill Density on Tensile and Flexural Strength
of 3D Printed Parts. J. Eng. Appl. Sci..

[128] Turaka S., Jagannati V., Pappula B., Makgato S. (2024). Impact of
Infill Density on Morphology and Mechanical Properties of 3D Printed
ABS/CF-ABS Composites Using Design of Experiments. Heliyon.

