# Supplementary material for: Optimization of the Mechanical Response in MEX Additive Manufacturing of Thermoplastic Polyimide (PI): The Impact of Key Process Control Settings
Source: ACS Omega. 2025 Nov 5;10(45):54764–80. doi: 10.1021/acsomega.5c08277 (PMC12631664; doi:10.1021/acsomega.5c08277)
Supplement: Supplementary file 1 [file ao5c08277_si_001.pdf]

Optimization of the mechanical response in MEX additive manufacturing of thermoplastic Polyimide (PI): The impact of key process control settings

Markos Petousis [a], Nikolaos Mountakis [a], Anastasios Zavos [b], Ioannis Ntintakis [a], Amalia Moutsopoulou [a], Maria Spyridaki [a], Nektarios K. Nasikas [c], Emmanuel Maravelakis [d], Nectarios Vidakis [a*]

[a] Department of Mechanical Engineering, Hellenic Mediterranean University, Heraklion 71410, Greece, markospetousis@hmu.gr; mountakis@hmu.gr; ntintakis@hmu.gr; amalia@hmu.gr; mspyridaki@hmu.gr; vidakis@hmu.gr

[b] Department of Mechanical Engineering and Aeronautics, University of Patras, Patras, 26504, Greece, zavos@upatras.gr

[c] Division of Mathematics and Engineering Sciences, Department of Military Sciences, Hellenic Army Academy, 16673 Vari, Attica, Greece, nasikas@sse.gr

[d] Department of Electronic Engineering, Hellenic Mediterranean University, Chania 73133, Greece, marvel@hmu.gr

* Corresponding author, E-mail: vidakis@hmu.gr (Nectarios Vidakis), Tel.: +302810379227

S.1. Taguchi design of experiments and regression modeling

Every factor possesses a DOF that can be found by subtracting one from the number of factor levels [1] and should not exceed the value of the DOF of the selected orthogonal array. The independent parameters for the dependent objective functions were distributed based on their importance [2]. In addition, a set of parameters is created that is capable of producing the best- and worst-case scenarios.

In the calculation procedure of the Taguchi method, the signal-to-noise (S/N) ratios for each investigated factor, the calculation of delta values from S/N ratios, and the determination of the order of each factor are included. Three forms define the S/N ratio [3] namely:

Larger the better [4]:

$$S/_{N} = -10\log\left(\frac{1}{n}\sum_{i=1}^{n}\frac{1}{Y_i^2}\right) \quad (1)$$

Smaller the better [5]:

$$S/_{N} = -10\log\left(\frac{1}{n}\sum_{i=1}^{n}Y_i^2\right) \quad (2)$$

Nominal the best [6,7]:

$$ {}^{S}\!/_{N} = 10 \log\left(\frac{\mu^2}{\sigma^2}\right) \tag{3} $$

Where:

- μ represents means
- σ represents the standard deviation.
- Yi represents the resulting value for the i_{th} objective function

The delta values were calculated by the difference between the maximum and minimum S/N values of each parameter and then ranked. The most effective parameter is represented by the largest delta value, and the rank is obtained from this order.

During the regression modeling, the calculation steps are as follows:
The total sum of squares (SS_T) was calculated using [8,9]:

$$ SS_T = \sum_{i=1}^{N} (Y_i - \overline{Y})^2 \tag{4} $$

where N is the number of cases in the orthogonal array, Y_i is the experimental and numerical results for the i_{th} experiment, and

$$ \overline{Y} = \frac{1}{N}\sum_{i=1}^{N} Y_i \tag{5} $$

The total sum of the squared deviations SS_T consists of the sum of the squared error, $SS_{e,,}$ and the sum of the squared deviations SS_P due to each process parameter; therefore, SS_P was defined as [8,9]

$$ SS_P = \sum_{j=1}^{t} \frac{(SY_j)^2}{t} - \frac{1}{N}\left[\sum_{i=1}^{N} Y_i\right]^2 \tag{6} $$

where P is one of the parameters, j is the level number of parameter P, t is the repetition of each level of parameter P, and SY_j is the sum of the experimental results involving parameter P and level j. The sum of squares from the error parameter SS_e is [8,9]:

$$ SS_e = SS_T - SS_A - SS_B - SS_C - SS_D - SS_E \tag{7} $$

The total degree of freedom was $D_T = N - 1$, and the degree of freedom of each tested parameter was $D_P = N - 1$. The variance of the parameters tested was $V_P = SS_P/D_P$. The F-value for each

design parameter is simply the ratio of the mean of squares deviations to the mean of the squared error, $F_P = V_P/V_e$. The percentage contribution ρ is calculated as follows [8,9]:

$$\rho_P = \frac{SS_P}{SS_T} \tag{8}$$

Additionally, two regression models were applied: the Reduced Quadratic Regression Model (RQRM) and the Linear Regression Model (LRM). The equations used are as follows:
The Linear Regression Model (LRM) for each response is calculated:

$$Y_k = a_k + \sum_{i=1}^{n} b_{i,k} x_i + e_k \tag{9}$$

The Reduced Quadratic Regression Model (RQRM) for each response is calculated:

$$Y_k = a_k + \sum_{i=1}^{n} b_{i,k} x_i + \sum_{i=1}^{n} c_{i,k} x_i^2 + e_k \tag{10}$$

Where:

- k represents the response output (i.e., Tensile Strength – σ_B^T, Tensile Yield Strength - s_Y^T , Tensile Modulus of Elasticity - E^T, Tensile Toughness T^T)
- a is the constant value
- b is the coefficients of the linear terms
- c is the coefficients of the square terms
- d is the coefficients of the two-way interaction terms
- e is the error and x_i is the five (n=5) control parameters (i.e., Raster Orientation - RO, Hot-end Temperature - HT, Printhead Velocity - PV, Internal Fill Ratio - IFR, Deposition Width - DW)

S.2. Experimental results

Table S1. Measured σ_B^T, σ_Y^T, E^T, T^T for each experimental run and five replicas per run

A/A	Run	$\sigma_B^T(MPa)$	$\sigma_Y^T(MPa)$	$E^T(MPa)$	$T^T(MJ/m^3)$
1		55.55	52.87	239.59	6.63
2		53.25	50.98	199.58	6.84
3	1	51.22	48.76	223.17	6.05
4		46.94	44.03	209.27	5.57
5		45.22	42.19	182.01	5.60
6	2	66.05	63.58	250.15	8.29
7		57.34	54.60	241.38	6.93

8		59.50	56.23	232.36	7.33
9		56.93	53.90	217.32	7.13
10		55.36	51.57	226.36	6.76
11	3	62.15	60.06	242.67	8.03
12		59.73	57.70	221.70	8.00
13		57.03	54.39	245.54	7.07
14		58.70	55.91	228.93	7.59
15		55.41	52.36	223.38	7.04
16	4	53.84	53.48	201.05	7.65
17		42.88	41.03	192.46	5.50
18		38.29	36.13	180.12	5.05
19		47.96	46.02	204.54	6.18
20		37.13	34.95	153.77	5.06
21	5	57.25	54.64	193.32	8.01
22		51.98	49.03	221.27	6.28
23		47.64	44.38	175.63	6.20
24		39.68	36.26	149.94	5.23
25		39.74	36.31	150.92	5.24
26	6	66.69	63.36	271.31	8.16
27		68.31	65.09	264.47	8.65
28		61.98	58.36	268.89	7.37
29		60.16	56.24	283.42	6.98
30		60.04	56.28	256.48	7.20
31	7	41.09	40.43	151.43	5.76
32		32.59	30.60	152.68	4.04
33		28.95	26.99	113.21	3.87
34		38.65	37.28	172.50	4.72
35		31.62	29.78	132.36	4.07
36	8	58.98	57.23	252.16	7.54
37		55.83	53.34	274.00	6.65
38		59.77	57.85	286.61	7.14
39		57.87	55.40	285.50	6.84
40		60.02	57.63	279.47	7.27
41	9	50.95	44.17	191.25	7.23
42		46.66	43.04	216.27	5.92

43		41.38	37.28	191.66	5.37
44		38.98	34.80	163.31	5.27
45		42.19	38.34	168.55	5.74
46		48.11	46.51	169.39	7.27
47		34.71	32.40	135.03	4.88
48	10	34.98	32.61	153.93	4.74
49		43.63	41.85	196.35	5.59
50		37.18	35.04	162.73	4.97
51		46.25	44.05	233.19	5.13
52		44.82	42.12	240.23	4.80
53	11	42.98	40.26	233.22	4.60
54		41.81	39.01	233.38	4.44
55		35.13	32.28	179.89	4.02
56		48.96	47.10	193.77	6.06
57		46.05	43.12	220.29	5.02
58	12	45.38	42.69	211.55	5.00
59		48.68	46.23	228.52	5.33
60		41.89	39.02	213.56	4.56
61		33.28	32.72	121.02	5.83
62		22.42	21.30	86.54	3.86
63	13	27.85	26.97	114.62	4.51
64		21.41	19.92	89.41	3.67
65		19.91	18.39	80.82	3.53
66		36.10	35.31	176.97	4.17
67		28.52	26.80	162.75	3.02
68	14	29.76	28.36	165.57	3.17
69		28.82	27.29	167.60	3.03
70		32.41	31.00	194.56	3.30
71		55.32	50.94	246.64	6.33
72		62.25	59.01	263.90	7.34
73	15	60.06	56.21	278.50	6.71
74		61.04	57.75	271.96	6.96
75		62.19	58.51	275.42	7.11
121	16	55.01	51.91	235.44	6.91
122		56.47	53.13	235.51	7.18

123		54.58	51.54	251.60	6.60
124		59.95	57.56	269.60	7.36
125		57.24	54.54	273.73	6.80
	Min:	19.91	18.39	80.82	3.02
	Max:	68.31	65.09	286.61	8.65
	Average:	47.56	44.98	206.86	5.94

Table S2. Measured σ_B^T, σ_Y^T, E^T, T^T for the five replicas of the Confirmation experimental runs

A/A	Run	$\sigma_B^T(MPa)$	$\sigma_Y^T(MPa)$	$E^T(MPa)$	$T^T(MJ/m^3)$
1		56.73	46.04	252.52	5.79
2		61.02	54.24	264.13	6.58
3	17	65.57	52.99	241.79	6.13
4		60.49	49.64	271.18	6.04
5		66.60	57.03	285.83	5.46
6		45.26	35.89	189.96	4.45
7		43.17	40.73	180.67	5.21
8	18	40.71	38.36	173.98	4.92
9		42.13	42.75	183.42	4.70
10		45.43	41.94	193.03	4.62
	Min:	40.71	35.89	173.98	4.45
	Max:	66.60	57.03	285.83	6.58
	Average:	52.71	45.96	223.65	5.39

References

(1) Ning, M.; Mengjie, S.; Mingyin, C.; Dongmei, P.; Shiming, D. Computational Fluid Dynamics (CFD) Modelling of Air Flow Field, Mean Age of Air and CO2 Distributions inside a Bedroom with Different Heights of Conditioned Air Supply Outlet. *Appl. Energy* **2016**, *164*, 906–915. https://doi.org/10.1016/j.apenergy.2015.10.096.

(2) Arslanoglu, N.; Yigit, A. Investigation of Efficient Parameters on Optimum Insulation Thickness Based on theoretical-Taguchi Combined Method. *Environ. Prog. Sustain. Energy* **2017**, *36* (6), 1824–1831. https://doi.org/10.1002/ep.12628.

(3) Simpson, J. R. Taguchi Techniques for Quality Engineering. *J. Qual. Technol.* **1996**, *28* (4), 487–489. https://doi.org/10.1080/00224065.1996.11979713.

(4) Bademlioglu, A. H.; Canbolat, A. S.; Yamankaradeniz, N.; Kaynakli, O. Investigation of Parameters Affecting Organic Rankine Cycle Efficiency by Using Taguchi and ANOVA Methods. *Appl. Therm. Eng.* **2018**, *145*, 221–228. https://doi.org/10.1016/j.applthermaleng.2018.09.032.

(5) Arslanoglu, N.; Yigit, A. Experimental Investigation of Radiation Effect on Human Thermal Comfort by Taguchi Method. *Appl. Therm. Eng.* **2016**, *92*, 18–23. https://doi.org/10.1016/j.applthermaleng.2015.09.070.

(6) Soni, A.; Patel, R. M.; Kumar, K.; Pareek, K. Optimization for Maximum Extraction of Solder from Waste PCBs through Grey Relational Analysis and Taguchi Technique. *Miner. Eng.* **2022**, *175*, 107294. https://doi.org/10.1016/j.mineng.2021.107294.
(7) Palanikumar, K. Experimental Investigation and Optimisation in Drilling of GFRP Composites. *Measurement* **2011**, *44* (10), 2138–2148. https://doi.org/10.1016/j.measurement.2011.07.023.
(8) Ri, R. H.; Yang, W.-C. Optimization of the Metal Injection Molding Process with 3l6L Stainless Steel Powder and Influence Analysis of Process Parameters Using the Taguchi-MADM-Based Hybrid Method. *ACS Omega* **2025**, *10* (1), 985–994. https://doi.org/10.1021/acsomega.4c08201.
(9) Mishra, S. S.; Mohapatra, T.; Sahoo, S. S.; Mishra, P. Overall Performance Investigation and Optimization of a Multi-Fuel Operated Compression Ignition Engine Using Coupled Taguchi and Grey Relational Analysis. *ACS Omega* **2022**, *7* (37), 33216–33232. https://doi.org/10.1021/acsomega.2c03566.
